# Liposomal Drug Delivery in Ocular Therapy: Strategies for Enhancing Corneal Penetration and Bioavailability

**DOI:** 10.3390/cells15171534

**Published:** 2026-08-26

**Authors:** Palak Mehta, Erik Moore, Alekha Dash, Surabhi Shukla

**Affiliations:** Department of Pharmacy Sciences, School of Pharmacy and Health Professions, Creighton University, 2500 California Plaza, Omaha, NE 68178, USA; palakmehta@creighton.edu (P.M.); erikmoore@creighton.edu (E.M.); alekhadash@creighton.edu (A.D.)

**Keywords:** liposomes, ocular drug delivery, corneal permeation, cationic, surface modification, bioavailability, PEGylation, nanocarriers, ophthalmic, mucoadhesive, ligand, dendrimer

## Abstract

Vision impairment affects approximately 2.2 billion people worldwide, with glaucoma, age-related macular degeneration, fungal keratitis, and diabetic retinopathy among the leading causes of preventable blindness. Effective pharmacotherapy remains severely constrained by the eye’s multilayered barrier architecture. Tear film, the corneal epithelium, the blood–aqueous barrier, and the blood–retinal barrier collectively restrict conventional topical drug bioavailability to less than 5% of the administered dose. Liposomal drug delivery systems have emerged as a clinically translatable platform capable of overcoming these barriers through targeted surface modification. This review provides a brief introduction to ocular barriers to drug delivery and transport and critically examines numerous surface-modification strategies applied to liposomal carriers to enhance corneal permeation and ocular bioavailability of drugs. It highlights the advantages and disadvantages of each modification strategy, as well as the convergent mechanism of liposomal surface modification in overcoming ocular barriers, and provides a comparative analysis of different surface-modification strategies of liposomes in terms of safety, efficacy and corneal retention. Additionally, it describes challenges associated with liposomal ophthalmic formulations in industrial scaling up. The review also sheds light on some FDA-approved liposomal ophthalmic products, active clinical trials on liposomal formulations, and relevant patents, demonstrating the potential benefits of liposomal drug delivery in the treatment of ocular disorders.

## 1. Introduction

Vision loss constitutes a major global public health issue. According to the World Health Organization’s 2023 estimates, approximately 2.2 billion people worldwide suffer from near or distance vision impairment, with nearly half of these cases being either preventable or treatable. Among these, the principal causes of distance vision loss include cataracts (5%), uncorrected refractive errors (4%), age-related macular degeneration (AMD; 0.4%), glaucoma (0.35%), and diabetic retinopathy (0.2%) [1].

Near vision impairment, predominantly caused by presbyopia, affects approximately 826 million individuals globally [2]. Vision loss is also a major public-health issue that has a substantial economic cost, which translates to the estimated global yearly loss of productivity in the form of 411 billion USD. The burden of ocular conditions is especially high in the United States, where recent statistics show that approximately 90 million adults aged 40 years and above (close to 60% of the total adult population) are visually impaired or have ocular disorders [1].

The human eye is a very specialized and sensitive organ that is surrounded by a range of structural and physiological barriers that ensure that the visual axis is not infected or inflamed [3]. The eye is anatomically divided into anterior and posterior parts. The anterior part includes the cornea, conjunctiva, iris, ciliary body and lens. The posterior part includes the vitreous humor, retina, choroid, sclera and optic nerve [4]. Drug delivery to ocular tissues is impeded not only by these static structural barriers but also by dynamic mechanisms, including tear turnover, blinking, nasolacrimal drainage, choroidal and conjunctival blood flow, lymphatic drainage, and active efflux transporters. All of these barriers act collectively to limit therapeutic drug concentrations at a target site in the eye [5].

Current ocular drug delivery routes include topical, systemic, intraocular, and periocular administration [6,7]. Topical formulations such as eye drops and ointments remain the most widely used treatment modalities, owing to their convenience and patient acceptability. However, their clinical utility is compromised by rapid precorneal clearance through tear drainage, nasolacrimal duct elimination, nonproductive conjunctival absorption, and the intrinsically low permeability of the corneal membrane [8,9]. As a result, topically instilled drugs remain in the tear film for only approximately 1 to 3 min, achieving bioavailability typically below 5% [10,11]. While intravitreal injections and FDA-approved implantable systems have partially addressed posterior segment delivery challenges, the overall therapeutic options remain limited relative to other routes of administration. These constraints underscore the urgent need for advanced drug delivery approaches capable of extending corneal contact time, enhancing permeation, and improving both anterior and posterior-segment bioavailability.

Nanocarrier-based delivery systems have demonstrated considerable promise in overcoming ocular barriers, offering improved drug retention, permeation, and sustained release [12]. Among these, liposomes stand out as the most biocompatible, biodegradable vesicles with a unique ability to encapsulate both hydrophilic and lipophilic drugs, making them particularly well suited for ocular applications [13]. More importantly, their surface properties can be tailored to improve interaction with the ocular surface and targeting. Cationic liposomes exhibit enhanced adsorption with the polyanionic corneal and conjunctival epithelia, prolonging drug retention and improving absorption [14]. PEGylation increases colloidal stability and reduces enzymatic degradation and clearance [15,16,17,18]. Mucoadhesive coatings based on chitosan and related polymers transiently modulate epithelial tight junctions and enhance corneal residence time [19]. Furthermore, ligand-functionalized nanoparticles containing targeting moieties, such as penetratin [20], cyclic peptide cRGD [21], and hyaluronic acid [22], facilitate receptor-mediated endocytosis and tissue-specific delivery superior to non-targeted systems.

This review provides a detailed and critical examination of surface-modified liposomal strategies aimed at enhancing corneal penetration and ocular bioavailability. In addition to formulation design principles, the translational potential of these systems is assessed through a discussion of FDA-approved liposomal ophthalmic products, active clinical trials, and relevant patents, demonstrating the advancing role of modified liposomes as clinically viable and patient-centered platforms in ocular therapeutics.

## 2. Barriers to Ocular Drug Delivery

**Figure 1:**
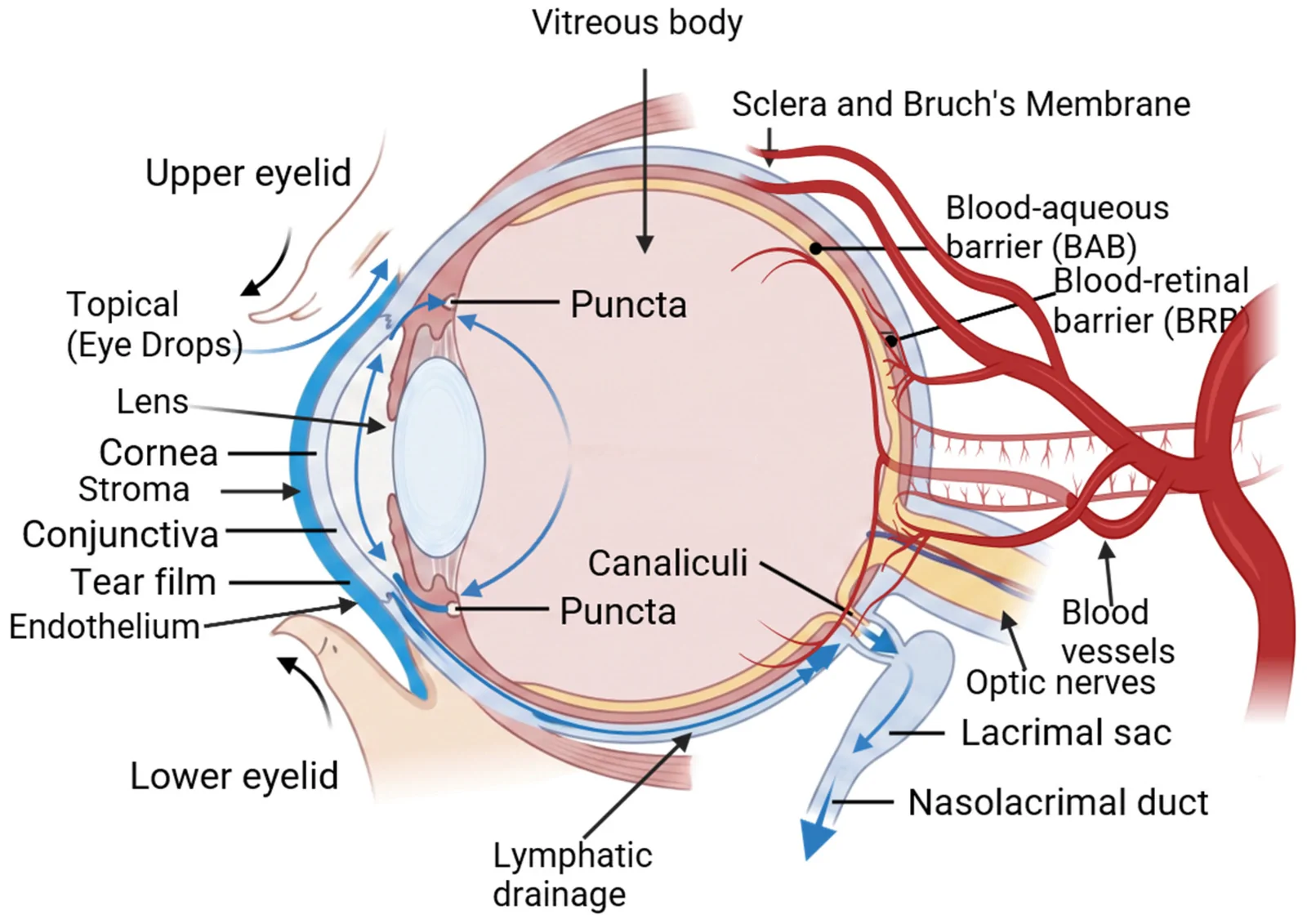
Depicting the eye anatomy associated with drug delivery. Created in BioRender. Moore, E.D. (2026): https://app.biorender.com/illustrations/6a777961de3ceba64f735776 (Made using BioRender).

Effective ocular drug delivery is governed by an interplay between static, dynamic, and intraocular barriers that vary by anatomical segment. Table 1 provides a structured overview of these barriers in ophthalmic delivery, and Figure 1 illustrates the eye’s anatomy associated with ophthalmic delivery.

### 2.1. Anterior Segment Barriers

#### 2.1.1. Tear Film and Blinking

The tear film is the first and most dynamic barrier that topically administered drugs encounter. It is about 3 mm thick and contains three functional layers: the outer lipid layer, which assists in preventing evaporation, the middle aqueous layer, and the inner mucous layer, which keeps the surface hydrated and prevents debris from entering the cornea [23,24].

The normal tear flow continuously washes drugs away from the eye, causing drug loss at a rate much higher than corneal absorption. In addition, irritation caused by formulation excipients or pH changes can trigger reflex tearing, resulting in the loss of more than 85% of the administered dose before it reaches the cornea [25]. These factors rapidly dilute the drug on the eye surface, resulting in very low ocular penetration and bioavailability, typically only 0.1–5% of the administered dose [26].

The amount of the tear film is continuously replenished with a flow rate of about 1 μL/min and a total volume of only 7–9 μL, resulting in full replacement in minutes [27]. A coordinated network of ocular surface sensory nerves, as well as the scentral nervous system and associated ocular glands, is known as the lacrimal functional unit, which regulates this process [28]. Tear film turnover is increased even more by blinks, which happen at a rate of 2 to 50 blinks per minute [29]. While essential for corneal integrity and visual clarity, these mechanisms significantly reduce the precorneal contact time available for drug absorption [30].

#### 2.1.2. Nasolacrimal Drainage

The nasolacrimal drainage system keeps the eye in homeostasis, and the residence time of topical drugs is influenced by this system. Its main action is the removal of tears, starting at the puncta. Puncta are small openings at the midpoint of both eyelids continuing on through the canaliculi, which culminate in the common canaliculus, and then empty into the lacrimal sac, followed by the inferior meatus of the nasal cavity [31].This frequent clearance presents significant drug delivery issues.

Up to 80% of an eye drop is lost in 15–30 s of application [32,33], radically decreasing bioavailability and requiring frequent dosing. The production of tears, the frequency of blinks, and the volume of drops modulate the rate of drainage [34]. Larger eye drop volumes in general increase loss by overflow and reflex tearing [35,36]. Substances entering this system can also be absorbed nasally, can enter the systemic circulation and can have off-target effects, especially for substances with high mucosal permeability [37]. To overcome these challenges, viscosity-enhancing agents are used to increase the drug’s residence time on the eye surface, while punctal occlusion helps reduce tear drainage and maintain therapeutic drug levels [1,12,38].

#### 2.1.3. Cornea

The avascular transparent outermost eye structure, the cornea, acts as the main point of static resistance to topical drug delivery to the eye and plays a very important refractive role. Anatomically, it is made up of three cellular layers, namely the lipophilic epithelium, hydrophilic stroma, and lipophilic endothelium, separated by two acellular membranes, which are the Bowman layer and the Descemet membrane, and each has a specific contribution to its barrier role. The corneal epithelium is composed of five to seven layers of lipid-rich cells interconnected by tight junctions and desmosomes. This is the most restrictive layer, permitting passage predominantly to small, non-polar compounds with a Log *p* value between 2 and 4 while largely excluding polar molecules and macromolecules [10,39,40,41,42,43].

Beneath the epithelium lies Bowman’s layer, an acellular collagenous structure approximately 8–12 μm thick composed primarily of type I and type III collagen fibrils. Although historically considered to offer minimal resistance to small molecules, its role in macromolecular transport, as investigated extensively by Wilson and colleagues at the Cleveland Clinic, remains an active area of inquiry [43]. Notably, Bowman’s layer thins by approximately one-third between the ages of 20 and 80 years and exhibits poor regenerative capacity, factors that may warrant greater consideration in the design of penetrating drug delivery systems [10,44].

The corneal stroma, accounting for approximately 90% of total corneal thickness (500 µm), is composed of highly hydrated type I collagen and supports the diffusion of hydrophilic drugs. Its viscosity, approximately 1.5-fold higher than that of water, does not typically represent a rate-limiting barrier for polar molecules. Maurice and Mishima demonstrated that compounds with molecular weights up to 500 kDa can diffuse through stromal tissue [45,46,47,48]. In contrast, the stroma significantly resists the permeation of non-polar compounds and may also act as a depot for drugs that fail to traverse the epithelium, with important pharmacokinetic consequences [9,49,50].

Descemet’s membrane, situated at the stroma–endothelium interface and composed of type IV and VIII collagen, offers limited resistance to small-molecule transport but can impede the passage of macromolecules and particulates introduced into the stroma. The innermost lipophilic layer, the corneal endothelium, is responsible for the maintenance of corneal hydration and transparency by the activity of Na^+^/K^+^-ATPase pumps, bicarbonate transporters, aquaporins and intercellular tight junctions [51,52]. Drug permeation through the endothelium is lower than through the epithelium, but it is generally sufficient for ocular drug delivery and is influenced by the drug’s molecular weight [43,53,54].

#### 2.1.4. Conjunctiva

The conjunctiva, while more permeable than the cornea due to its larger surface area and less restrictive epithelium, remains a significant barrier to ocular drug absorption. Conjunctival drug uptake often results in systemic absorption rather than intraocular delivery, contributing to both therapeutic loss and potential systemic side effects. The conjunctival vasculature and lymphatic drainage further hasten drug clearance from the ocular surface [5,35].

#### 2.1.5. Stroma

The stroma, a hydrophilic layer located in the middle of the cornea and posterior to Bowman’s layer, constitutes about 90% of the corneal thickness (up to 500 µm thick) [45,46]. The stroma is often described in the literature as exhibiting fluid-like characteristics with a viscosity of approximately 1.5-fold that of water [47]. For polar compounds, the stroma generally does not present a rate-limiting barrier. Maurice and Mishima [9] demonstrated that molecules with a molecular weight of up to 500 kDa can diffuse across the stromal tissue. However, for non-polar compounds, the stroma has been shown to impart significant barrier properties [49]. Furthermore, the stroma also exhibits a reservoir effect for drugs that successfully penetrate through the epithelium. It is worth noting that this phenomenon has significant implications [5,50].

#### 2.1.6. Endothelium

The second lipophilic barrier is the corneal endothelium, which is the innermost layer of the cornea and is important in the maintenance of corneal hydration, clarity and normal function. The endothelial cells of the cornea have a high density of Na^+^/K^+^ ATPase pumps on the basolateral membrane that actively move ions across the stroma to aqueous humor [55]. Bicarbonate transporters [56], aquaporins [57], water channels, and tight junctions also aid in the formation of an osmotic gradient, which controls fluid movement across the membrane. Even though the endothelium of the cornea is said to be more permeable than the corneal epithelium due to its leaky characteristic and directional transportation, the molecular weight also affects drug permeation [54]. This is a significant factor to be considered in the design of the ocular drug delivery system.

#### 2.1.7. Blood–Aqueous Barrier

The blood–aqueous barrier (BAB) is formed by tight junctions in the ciliary process’s non-pigmented epithelium, endothelial cells in the iris vasculature, and the inner wall endothelium of Schlemm’s canal. The tight junctions regulate paracellular transport, controlling the movement of ions and small molecules between adjacent cells. The BAB is not completely impermeable; instead, it serves as a specialized gateway for controlled molecular movement [58].

### 2.2. Posterior-Segment Barriers

Drug delivery to the posterior segment is governed by two principal intraocular barriers: the blood–aqueous barrier (BAB), formed by the ciliary epithelium and iris vasculature, and the blood–retinal barrier (BRB), comprising the tight junctions of retinal vascular endothelial cells (inner BRB) and the retinal pigment epithelium (outer BRB). Together, these barriers severely restrict the passage of systemically or topically administered drugs to the retina, choroid, and vitreous, necessitating invasive delivery routes such as intravitreal injection for the treatment of many posterior segment conditions [5,6]. The pharmacokinetic constraints specific to posterior segment delivery include rapid vitreous turnover, the extensive diffusion distances to the retina, and the absence of passive absorption from the anterior chamber. This has been systematically characterized by Geroski and Edelhauser, whose foundational work established the benchmarks for evaluating transscleral and intravitreal strategies [46].

#### 2.2.1. Vitreous Body

The vitreous is a gel-like fluid that is clear and is located between the lens and the retina. It consists primarily of water, type II, IX, and V/XI collagen, hyaluronic acid and additional extracellular matrix molecules. The positively charged nanomaterials have the ability to react with the negatively charged vitreous elements, which restricts diffusion, but the negatively charged nanoparticles, such as poly lactic-co-glycolic acid (PLGA) and human serum albumin, can freely penetrate the vitreous humor [59]. The vitreous also offers structural support and prevents eye shape distortion under intraocular pressure. The vitreoretinal interface is a wall that restricts the movement of substances into the retinal layers [60]. It includes: (1) the cortical vitreous, a 100–300 µm layer of collagen parallel to the inner limiting membrane (ILM); (2) the ILM, a layer consisting primarily of collagen type IV, laminin and fibronectin, a physical barrier; and (3) Müller cell footplates, glial cells that extend on the vitreous side of the retina.

#### 2.2.2. Sclera and Bruch’s Membrane

Choroid refers to a very vascular barrier that is between the retinal pigment epithelium (RPE) and sclera. It is approximately 200 µm in thickness with five layers: the Bruch membrane, the choriocapillaris, two vascular layers and the suprachoroidal layer [59,61]. It restricts the flow of hydrophilic drugs, whereas positively charged lipophilic drugs can be attached to the tissue and develop slow-release depots. Molecular size also influences the diffusion of drugs to the posterior segment. The thickness of the Bruch membrane is approximately 2–4 mm, and it consists of collagen and elastin fibers. The choriocapillaris includes extensively fenestrated capillaries that have 6–12 nm diameter pores that permit the passage of larger molecules [62]. The outer opaque part of the eye is called the sclera, and it is primarily made up of collagen fibers and proteoglycans, as well as glycoproteins. It is 0.5–1 mm thick, and the movement of drugs across the sclera varies with the molecular weight, size, charge, and lipophilicity. Hydrophilic drugs like methazolamide can pass through the sclera. The scleral proteoglycan matrix is negatively charged at a physiological pH, hence preferring the diffusion of negatively charged solutes [63].

#### 2.2.3. Conjunctival Blood Flow

The last dynamic barrier concerning ocular drug delivery is the conjunctival blood flow. The conjunctiva is a thin, colorless-pink mucous membrane, which covers the anterior part of the eye and the inside of eyelids. It is made of epithelial cells and connective tissue that is very rich in blood vessels. The conjunctival vascular network is an arterioles, capillaries, and venules system spread throughout the substantia propria, and is generally referred to as conjunctival blood flow [64]. With this rich vascularization, the conjunctiva takes on its typical pink appearance and is an important route of clearance of drugs. When a drug enters the conjunctiva, it may be taken up quickly into the bloodstream in the conjunctiva and then eliminated into the bloodstream. This accelerated clearance of the drug in the bloodstream decreases the local accumulation of drug at the ocular site and can also lead to an increased number of systemic side effects.

#### 2.2.4. Lymphatic Drainage

In contrast to the cornea, conjunctival blood flow and ocular lymphatic drainage have a strong limiting effect on conjunctival drug absorption. Once absorbed across the conjunctiva, the drugs can be swept away into the systemic circulation at a high rate by capillaries and lymphatic vessels, decreasing the bioavailability in the eye. Hydrophilic drug delivery is also hindered by tight junctions. Scleral permeability beyond the conjunctiva is determined by molecular size and charge. Thus, bigger and positively charged molecules exhibit the lowest permeation [5,65].

#### 2.2.5. Blood–Retinal Barrier

The blood–retinal barrier (BRB) is a very selective barrier that controls the transport of ions, proteins, water and drugs in and out of the retina. It is made of the outer BRB, which is made up of the choroid, Bruch membrane and the retinal pigment epithelium, and the inner BRB, which is made up of tight junctions between retinal capillary endothelial cells [66]. The combination of these barriers inhibits the permeation of drugs according to their molecular size, charge, and lipophilicity. Small hydrophilic molecules can enter the cell via paracellular routes, and lipophilic drugs tend to enter the cell via transcellular diffusion [67]. The BRB also harbors influx transporters and efflux pumps that control drug intake and excretion and are thus valuable targets to enhance retinal drug delivery [59].

## 3. Strategies for Enhancing Corneal Permeability and Ocular Bioavailability

**Figure 2:**
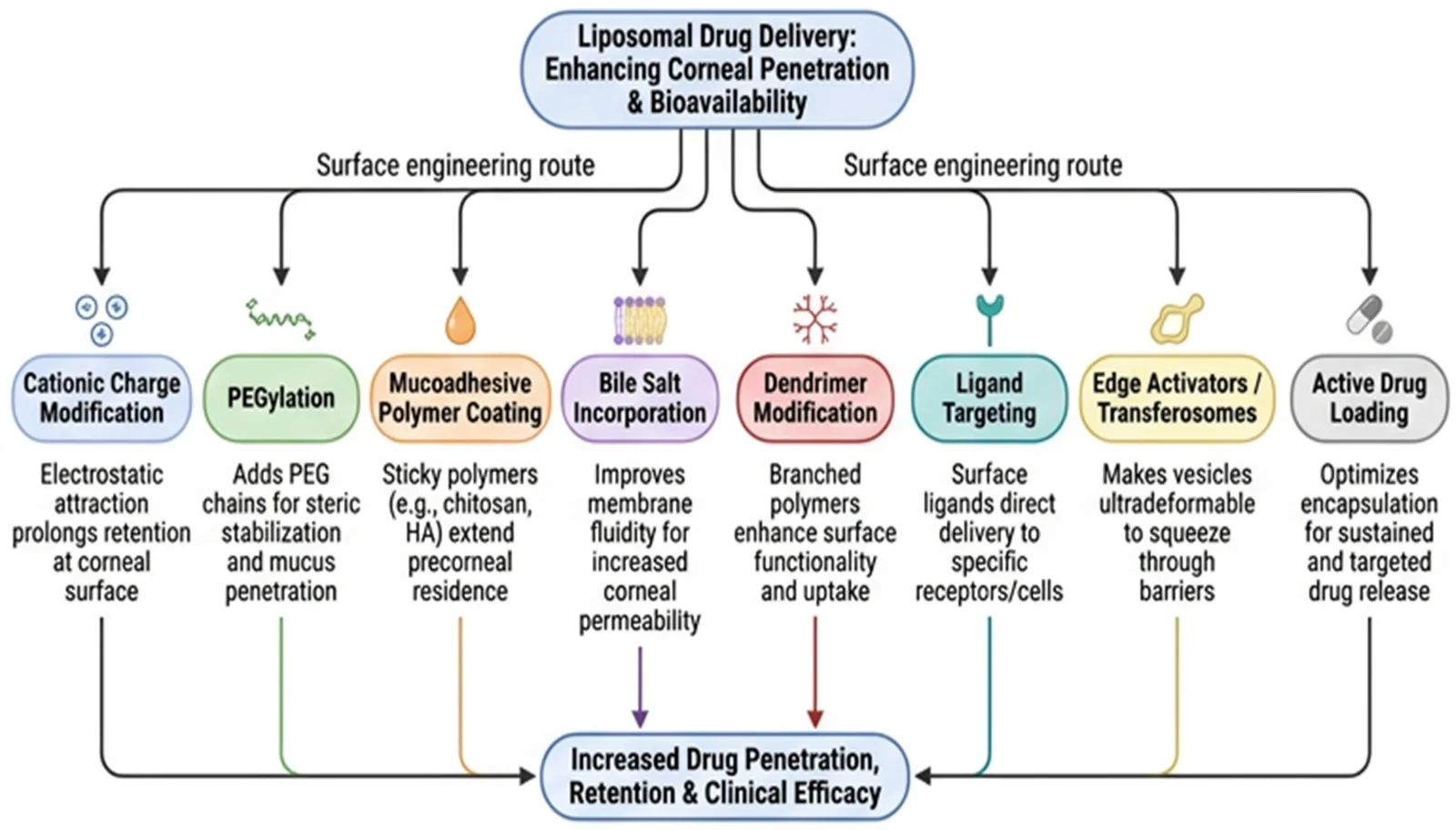
Strategies for enhancing corneal penetration and ocular bioavailability (Made using FigureLabs).

**Figure 3:**
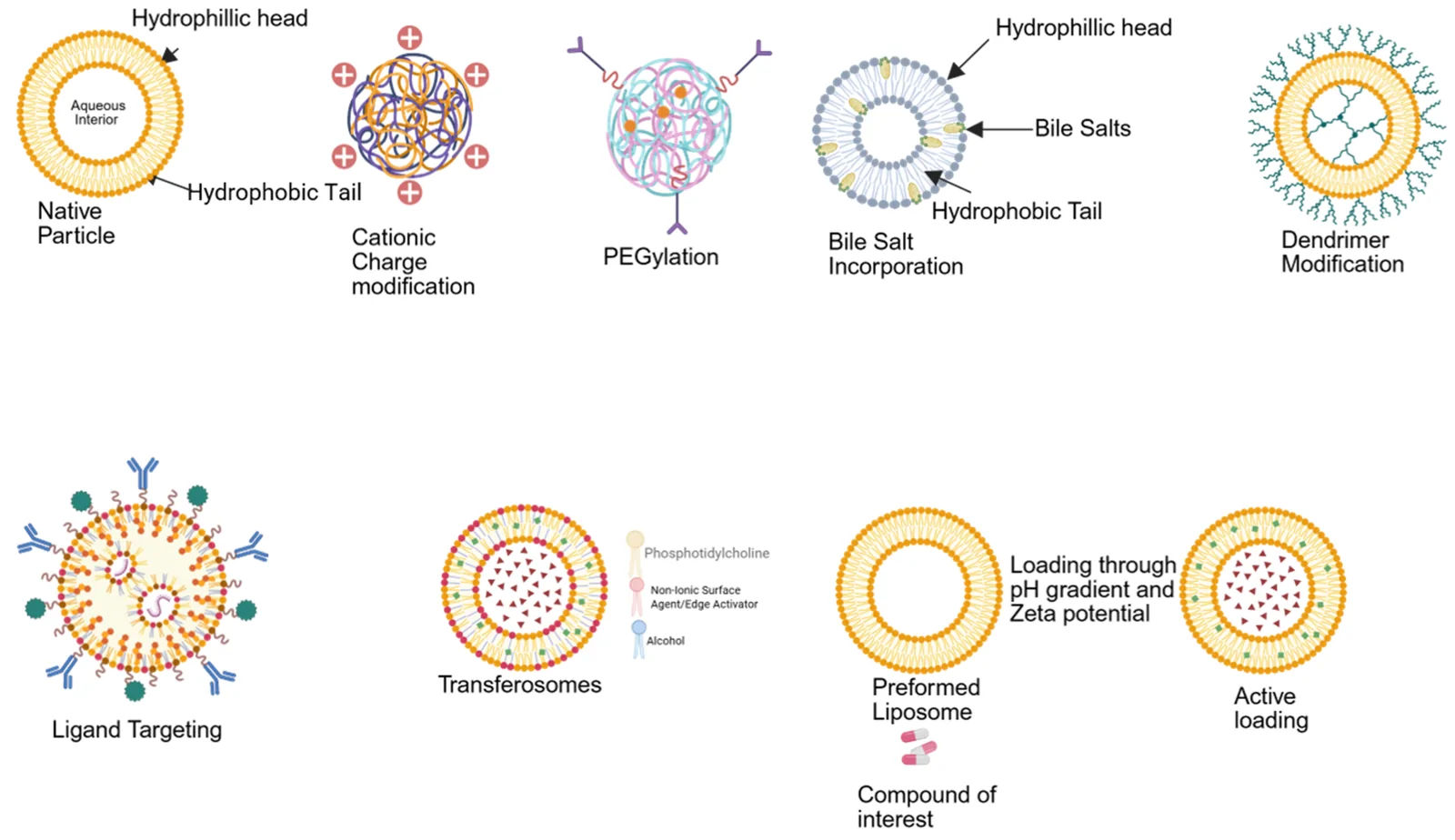
Comparative analysis of specific structural features of each surface-modified strategy. Created in BioRender. Moore, E.D. (2026) https://app.biorender.com/illustrations/6a777961de3ceba64f735776 (Made using BioRender).

Multiple surface modifications and formulation strategies have been designed to enhance the corneal permeability and ocular bioavailability of liposomal drug delivery systems. These approaches include cationic charge modification, which promotes electrostatic interaction with the negatively charged corneal surface and improves retention time, and PEGylation, which enhances steric stabilization and facilitates mucus penetration. Mucoadhesive polymer coatings with chitosan or hyaluronic acid further prolong precorneal residence. The incorporation of bile salt increases membrane fluidity, thereby improving corneal permeability. Additionally, dendrimer modification and ligand targeting enable enhanced cellular uptake and receptor-specific delivery. The incorporation of edge activators to form transferosomes increases vesicle deformability, allowing better penetration across ocular barriers, whereas active drug-loading techniques optimize encapsulation efficiency and sustain drug release. Collectively, as depicted in Figure 2, these complementary strategies contribute to improved drug penetration, prolonged retention, and enhanced therapeutic efficacy in ocular applications. Figure 3 depicts specific structural features of each surface-modified liposome.

### 3.1. Overview of Clinically Approved Liposomal Ophthalmic Formulations

There are many nanocarrier-based ocular drug products utilizing micelles and gel systems. However, the clinical translation of liposomal drug delivery systems in ophthalmology is best exemplified by one, the FDA-approved Visudyne^®^. Visudyne^®^ (verteporfin liposomal formulation) is indicated for photodynamic therapy in patients with predominantly classic subfoveal choroidal neovascularization (CNV) secondary to AMD, pathologic myopia, or presumed ocular histoplasmosis [59,68]. There are several commercially available products that may be sold under general product safety rules rather than as a regulated drug. Lacrisek^®^, a liposomal spray containing vitamins A and E, and Artelac Rebalance^®^, a liposomal eye drop formulation incorporating vitamin B12, PEG, and hyaluronic acid, are both commercially available eye products for the clinical management of dry eye disease [69,70].

The success of these products demonstrates the therapeutic viability of liposomal platforms in ophthalmology and provides a foundation upon which next-generation, surface-engineered systems are being developed and evaluated. The breadth of liposomal utility as pharmaceutical carriers across therapeutic areas was comprehensively reviewed by Torchilin, whose seminal work established the biophysical and pharmacokinetic rationale underpinning liposomal drug delivery [71]. Table 2 shows FDA-approved liposomal ocular drug products and some commercially available eye care products.

In the ocular context specifically, Meisner and Mezei provided early evidence that liposomal formulations could meaningfully improve precorneal drug retention and transcorneal absorption compared to conventional solutions [72]. More recently, Mishra et al. consolidated the clinical and preclinical evidence base for liposomal ophthalmic applications, reinforcing the position of liposomes as a preferred nanocarrier platform for ocular indications [73].

A pilot study conducted by researchers at the Singapore Eye Institute evaluated a liposomal latanoprost formulation (NCT01987323). In this study, six participants with elevated intraocular pressure received subconjunctival injections of the EggPC-based liposomal formulation to assess its safety and preliminary efficacy. Although the study has been completed, no results have been posted on ClinicalTrials.gov.

TLC399 (ProDex), a liposomal dexamethasone prodrug formulation, has also undergone clinical evaluation. A Phase 2 randomized, double-masked trial (NCT03093701) investigating two dose levels of TLC399 in subjects with macular edema secondary to retinal vein occlusion was completed in April 2019. POLAT-001, a liposomal latanoprost subconjunctival injection, has undergone clinical studies. A phase 2, open-label comparison of the Safety and Efficacy of Subconjunctival Liposome Latanoprost (POLAT-001) to Latanoprost Ophthalmic Solution in Patients with Ocular Hypertension and Primary Open-Angle Glaucoma was initiated in July 2015, and the study was completed in April 2016. A clinical interventional study (NCT03617315) was initiated for a crosslinked hyaluronic acid with liposome and crocin in the treatment of dry eye disease with moderate meibomian gland dysfunction. A total of 50 eyes from 25 adult patients were evaluated, all of whom wore silicone hydrogel contact lenses. A phase IV clinical trial (NCT04087733) was conducted to assess the therapeutic potential of a liposomal ozone-based solution (OZODROP^®^) in the preparation of the patient for cataract surgery by evaluating the reduction in bacterial colonization of the conjunctiva.

An early-phase I clinical investigation evaluated the safety and preliminary efficacy of subconjunctival administration of liposomal sirolimus for the treatment of moderate to severe dry eye disease (DED). The ocular-surface disease index is examined on a scale of 0 to 100, with the highest scores representing greater disability. Some liposomal ophthalmic formulations in the clinical stage are summarized in Table 3.

The development of the Intellectual Property (IP) portfolio for liposomal drug delivery to the eyes mirrors the growing commercial and scientific importance of the field. Selected patents, summarized in Table 4, illustrate some of the important formulation innovations and therapeutic targets.

### 3.2. Cationic Liposomes: Surface-Charge Modification

The electrostatic environment of the ocular surface is a key factor in drug absorption into the cornea and drug retention. The corneal and conjunctival epithelial surfaces are covered by mucin glycoproteins with negative charges, which have an electrostatic attraction effect towards positively charged delivery systems [14]. Cationic liposomes take advantage of it to obtain long-lasting adhesion to the ocular surface, longer precorneal residence, and better trans corneal permeation of the drug. This approach has been shown to work over a variety of classes of drugs. The first to demonstrate that positively charged liposomes had excellent corneal adsorption properties over neutral or anionic liposomes were Fresta et al. [14]. Lajunen et al. have shown that the transcorneal penetration of penicillin G could be enhanced up to 4-fold by cationic liposomes, which serves as a demonstration of the versatility of charge-mediated permeation enhancement [74].

The immunosuppressant tacrolimus (FK506) has been extensively studied in cationic liposomal formulations. In one study, the cationic liposomes were made using DOTAP and showed binding to the mucin layer on the surface of the cornea, and the corneal drug levels of tacrolimus were 3.59 ± 1.96 ng/mL at 5 min, 2.79 ± 1.07 ng/mL at 30 min, and 5.26 ± 1.75 ng/mL at 1 h, while the conventional eye drops yielded only 1.20 ± 0.43 ng/mL; in other words, the corneal drug accumulation was about 4–5 fold higher. Fluorescence imaging showed that the liposomes stayed on the ocular surface for 30 min, whereas the traditional solution was rapidly removed by tears. Most importantly, an equivalent therapeutic effect was obtained using a 4-fold lower dose (0.01 vs. 0.05 mg), showing the dose-saving effect of cationic surface-engineering modified liposomes [75]. Chen et al. have shown that cationic liposomes with 300 nm particle size and +30 mV surface-charge loaded with tacrolimus not only extended the ocular residence time and enhanced drug accumulation in the cornea but also resulted in a marked decrease in the levels of reactive oxygen species (ROS) and inflammatory markers that are associated with dry eye, compared to conventional formulations [75]. Surface-engineered cationic liposomes resulted in an AUC of approximately 30.29 μg·min/mL, which is more than 3-fold higher than the commercial eye drops with an AUC of 12.05 μg·min/mL. The reported C_max_ for this study was 3.87 μg/mL compared to 2.68 μg/mL for commercial drops, and the half-life and mean residence time were doubled [76]. Overall, the results mentioned above support cationic surface modification as one of the most practical and widely applicable methods to enhance corneal permeability and drug bioavailability in the eye. Gai et al. showed that the permeation of the cornea and the level of drug concentration in the aqueous humor were significantly higher in both in vitro and in vivo rabbit models when cationic liposomes containing ibuprofen were used compared to conventional formulations. This study demonstrates the generalizability of charge-mediated enhancement of corneal permeability and drug bioavailability in the eye to most classes of anti-inflammatory drugs [77].

The cfDNA-scavenging system refers to the body’s ability to detect, bind and remove circulating cell-free DNA (cfDNA) from the bloodstream. The body possesses a natural cfDNA-scavenging system that continuously recognizes and clears circulating cfDNA from the bloodstream to preserve immune homeostasis and prevent excessive inflammation. However, the limited capacity of this system under pathological conditions has inspired the development of biomimetic drug delivery platforms that mimic its DNA-binding and clearance functions. One such system was prepared as a cationic liposome and characterized to have a positive surface charge (+44.2 ± 2.3 mV) that enabled the system to possess high muco-adhesion and high precorneal retention and to prolong the persistence of corneal fluorescence for more than 15 min, as compared to the rapid clearance of the free dye solution. The engineered cationic liposomes (cL) exhibited a mean hydrodynamic diameter of 154.8 ± 1.5 nm, supporting efficient interaction with the ocular surface and corneal uptake.

Mechanistically, the surface-engineered cationic liposomes entered the cornea primarily through transcellular endocytosis. Importantly, the transient opening of tight junctions was reversible, as Zona Occludens-1 (ZO-1) expression returned to normal within 24 h, indicating no long-term disruption or tissue damage. The liposomes exhibited a DNA-binding affinity of approximately 83% at a nitrogen-to-phosphate (N/P) ratio of 10:1, where the N/P ratio represents the molar ratio of positively charged amine groups on the liposomes to negatively charged phosphate groups on DNA. Enhanced charge-mediated corneal permeation enabled the cationic liposomes to accumulate at the lesion site and efficiently scavenge excess cfDNA. Thus, improved delivery to the cornea was a key prerequisite for achieving the observed therapeutic efficacy [78].

The use of cationic surface-modified liposomal systems has already been tested for nanoemulsion platforms, such as the Novasorb technology (Novagali Pharma). This cationic nanoemulsion of the oil-in-water type with a ζ- potential of +20–+40 mV and a droplet size ranging from 150 to 300 nm has shown an electrostatic interaction with the anionic layer of the ocular mucous membrane that results in higher precorneal retention and higher transcorneal drug absorption. The spreading advantage was shown by the cationic emulsion (Cationorm), which spreads on the rabbit cornea in seconds with a near-zero contact angle. However, an anionic emulsion spreads very poorly with a contact angle of 42^º^ and a much smaller spreading coefficient. This resulted in an approximate 1.8-fold increase in corneal bioavailability of cyclosporine A, with a corneal C_max_ of 1372 vs. 748 ng/g, and AUC of 26,477 vs. 14,210 ng.h/g with no increase in systemic exposure. This permeation-enhanced bioavailability clinically resulted in a 49% vs. 30% decrease in dry eye symptom scores at Day 28 (*p* = 0.001), with much better tear-film break-up time (2.00 vs. 1.16 mm) at Day 28 (*p* = 0.015) compared to traditional artificial tears [79].

Dos Santos and coworkers incorporated besifloxacin into spermine-modified cationic liposomes (LP PC:SPM) and evaluated the influence of surface charge on passive corneal drug delivery. The positively charged LP PC:SPM had a mean hydrodynamic diameter of 175.4 ± 1.9 nm and zeta potential of +19.5 ± 1.0 mV, while the neutral control liposomes (LP PC) measured 177.2 ± 2.7 nm with a zeta potential of −5.7 ± 0.3 mV. The positive surface charge conferred mucoadhesive properties to LP PC:SPM, evidenced by a significant increase in hydrodynamic diameter upon mixing with mucin particles, an effect absent in the neutral LP PC formulation. When challenged against a simulated lacrimal flow of approximately 20 µL/min, only LP PC:SPM achieved statistically greater corneal drug retention compared to both the neutral liposomes and the commercial suspension Besivance (4.26 ± 0.74% vs. 2.65 ± 0.34% and 2.29 ± 0.15%, respectively). This improvement is attributed to the electrostatic interaction between the cationic liposomes and the negatively charged corneal surface. The positive charge effectively prolongs formulation residence time by resisting tear drainage, thereby enhancing drug-corneal contact and bioavailability [80].

Cortesi and coworkers illustrated that cationic liposomes are applicable not only to small-molecule drugs but also to macromolecular cargo such as viral antigens. Dimethyldioctadecylammonium bromide (DDAB)-containing cationic liposomes were prepared as carriers of herpes simplex virus type 1 (HSV-1) antigens for intraocular delivery. The formulations had a mean particle size ranging from 314.8 ± 12.9 to 327.6 ± 30.5 nm and a zeta potential ranging from +19.6 ± 1.2 to +26.3 ± 1.9 mV. The positive surface charge served two functions. First, it enhanced muco-adhesion to the anionic corneal surface, prolonging precorneal retention. Second, it acted as an immunological adjuvant. Encapsulation efficiency was approximately 100% for glycoprotein B (gB1s), whereas the poly-L-lysine-rich DTK peptides exhibited encapsulation efficiencies ranging from 28.6% to 32.4%.

The release profiles of liposomal formulations were comparable to those of free solution, confirming that encapsulation did not compromise antigen availability. Intraocular vaccination via conjunctival instillation of 4 µg gB1s/liposome preparation yielded 66.6% survival against an otherwise 100% fatal HSV-1 challenge in both vaccinated groups. Surviving animals produced detectable neutralizing antibody titers of 1:73 to 1:112. No evidence of viral reactivation from latency was observed. These findings demonstrate that cationic surface charge can prolong ocular exposure of macromolecular cargo sufficiently to elicit a protective immune response, extending the utility of cationic liposomes beyond small-molecule delivery to include mucosal vaccine administration [81].

Soni and Saini (2021) [82] presented a scientific DoE-based demonstration of the corneal permeation-enhancing effect of cationic surface charge by using liposomes containing DOTAP (EPCS: DOTAP, 1:1) with brimonidine tartrate, which is a poorly bioavailable antiglaucoma drug. The optimization of the formula diameter (150.4 nm), polydispersity index (0.203), zeta potential (+30.62 mV), and entrapment efficiency (55.17%) proved that it is the positive surface charge formed by DOTAP that causes intimate electrostatic adhesion to anionic corneal epithelium. This raises viscosity when in contact with ex vivo permeation across goat cornea, which directly quantified this bioavailability-enhancing effect. The cationic liposomal formulation achieved a steady-state flux of 17.63 ± 1.22 µg·cm^−2^·min^−1^ versus 8.67 ± 0.52 µg·cm^−2^·min^−1^ for drug solution. The reported apparent permeability coefficients were 1.011 ± 0.07, versus 0.497 ± 0.03 cm·min^−1^ for cationic liposomes and drug solution, respectively. This study represented a more than twofold enhancement in corneal drug permeation with a 50% shorter lag time (15 versus 30 min). A sustained 12-h drug release reaching 91.13% cumulative drug release was observed, which followed Higuchi’s square root kinetics [82].

Macwan et al. [83] reported that the liposomal composition optimization could significantly enhance the corneal administration of caspofungin. Optimized liposomal formulation with a 121.34 nm particle size, a PDI of 0.140, and a zeta potential of −55.4 mV with entrapment efficiency of 80.91% exhibited significantly increased permeation across excised goat cornea. A cumulative drug permeation of 98.56% was achieved after 12 h of incubation in comparison to 29.89% when using free drug solution. Moreover, the sustained release profile (>90% drug release during 12 h) promotes prolonged precorneal residence, which also leads to improved ocular bioavailability and efficacy [83].

Conclusion: Taken together, cationic surface-charge modification is the most extensively validated and reproducible strategy reviewed here, consistently improving corneal drug levels and AUC by approximately 2- to 5-fold across ten independent studies spanning eight structurally unrelated drug classes [14,74,75,76,77,78,79,80,81,82,83]. Its principal limitation is a charge-density-dependent cytotoxicity that becomes clinically relevant mainly for chronic, high-frequency dosing regimens, indicating that charge optimization, rather than charge maximization, should guide formulation design.

### 3.3. PEGylation and Stability Enhancement

PEGylation, the covalent or non-covalent attachment of polyethylene glycol (PEG) chains to the liposomal surface, represents a well-established strategy for improving the biopharmaceutical performance of liposomal systems. In the context of ocular drug delivery, PEGylation confers multiple advantages. It enhances colloidal stability, reduces protein adsorption and enzymatic degradation, and diminishes systemic and ocular clearance, thereby extending the circulating and tissue residence time of liposomal carriers [84].

Tavakoli et al. demonstrated in an in vitro bovine retinal explant model that both PEGylation and anionic surface modification improved retinal distribution of liposomes, and that small liposomes (~50 nm) successfully penetrated retinal tissue, whereas larger particles (~100 nm) showed minimal penetration. This highlights the combined importance of surface chemistry and nanoscale size in posterior-segment targeting [60]. In addition to improving stability, PEGylation has been shown to facilitate selective uptake by macrophages in inflamed tissues, a property of potential interest in the treatment of ocular inflammatory diseases [16,17,18].

Moiseev et al. [85] have shown that PEGylation, coupled with mucoadhesive functionalization, is a two-way approach to improve the ocular delivery of drugs due to its ability to improve the penetration of mucus and the retention of the precorneal drug. PEGylated liposomes (PEG2000) and maleimide-PEGylated liposomes (PEG2000-Mal) displayed similar drug release kinetics, with both releasing drug to greater than 100% cumulative release within 12–18 h, suggesting that PEGylation has no effect on drug availability. PEG is a mechanistic facilitator of paracellular transport, which temporarily relaxes epithelial tight junctions, thereby increasing permeation. The introduction of maleimide groups confers covalent interactions with mucin’s thiol groups, greatly increasing muco-adhesion.

This was numerically confirmed in retention studies, where all formulations showed rapid washout from the cornea within 5 min. However, on conjunctival tissue, LPEG2000-Mal demonstrated significantly higher retention compared to conventional liposomes and PEGylated systems. This indicates improved mucosal adherence and prolonged residence time on the conjunctiva, which retained its adhesion in a statistically superior manner within the 30 min study period. It is important to note that the increased retention is functionally important, as the conjunctiva is 8.6 ± 4.4 times more permeable than the cornea, thus having a stronger ability to absorb drugs with an increase in residence time. Taken together, this synergistic alliance of permeation enhancement through PEGs and muco-adhesion through maleimides forms an excellent combination to enhance the ocular bioavailability, due to increased tissue permeability and long-term retention on the surface [85].

In a study by Abdul Nasir et al. [86] the ocular tissue distribution of PEGylated and non-PEGylated liposomes was evaluated following topical administration in rats using the lipophilic fluorescent dye 1,1′-Dioctadecyl-3,3,3′,3′-tetramethylindocarbocyanine perchlorate (DiI) as a tracer. Fluorescence microscopy was used to assess dye distribution in the cornea, ciliary body, and retina at various time points after instillation.

Non-PEGylated liposomes exhibited a significantly greater fluorescence intensity in the cornea at 2 and 5 min post-instillation, indicating faster initial ocular-surface permeation. Similarly, fluorescence intensity in the ciliary body was significantly higher for non-PEGylated liposomes at 5 min, suggesting more rapid distribution to anterior ocular tissues.

In the retina, non-PEGylated liposomes showed higher fluorescence intensity at 5 min, whereas PEGylated liposomes demonstrated significantly greater retinal fluorescence intensity at 10 min (26.87 ± 7.44 vs. 18.45 ± 6.51 arbitrary fluorescence units; *p* < 0.05), indicating prolonged retention and sustained distribution to posterior ocular tissues. The authors attributed this behavior to the stealth properties conferred by PEGylation, which reduces uptake by phagocytic cells and extends residence time within ocular tissues. Overall, the study suggested that, while PEGylation may slow the initial permeation of liposomes across ocular barriers, it can enhance retention and sustained delivery to deeper ocular tissues, including the retina [86].

Jin et al. [87] incorporated D-α-tocopheryl polyethylene glycol succinate (TPGS), an amphiphilic vitamin E derivative, into liposomes to enhance membrane interaction and ocular drug transport. The optimized TPGS-modified brinzolamide-loaded liposomes (T-LPs/Brz) demonstrated a high encapsulation efficiency (95.41 ± 3.03%) and a nanoscale particle size (96.87 ± 4.43 nm), supporting excellent drug-loading capacity and colloidal stability.

TPGS modification significantly enhanced transcorneal transport. The apparent permeability coefficient (Papp) increased by 10.2-fold compared to the commercial formulation and 1.38-fold compared to conventional liposomes. Overall, corneal permeation improved by approximately 2- to 5-fold.

Moreover, it was found that there was an increase in precorneal retention, and the drug concentration increased 3.18 times compared to the commercial suspension at 2–3 h after administration, which means that residence time was improved and absorption was enhanced. TPGS-modified liposomes showed pharmacodynamic effects of a reduction in intraocular pressure of up to 35.17 in 3–11 h. This is due to the permeation-enhancing and P-glycoprotein inhibitory effects of TPGS, which increase drug delivery to the corneal barrier and decrease efflux, eventually enhancing bioavailability [87].

Conclusion: Unlike cationic modification, PEGylation alone does not consistently improve early corneal permeation and, in some studies, reduces it relative to non-PEGylated liposomes [86]; its benefit is instead concentrated in prolonged posterior-segment retention and reduced clearance. This time- and tissue-dependent profile explains why PEGylation is most effective as a complementary modification (e.g., paired with maleimide-mucoadhesive functionalization [85], rather than as a stand-alone permeation enhancer.)

### 3.4. Mucoadhesive Polymer Coatings

The mucoadhesive surface coatings are an alternative approach to providing extended precorneal residence time of liposomes by binding to the mucin layer of the tear film and conjunctiva. Chitosan and Hyaluronic acid (HA) are two polymers that have been extensively tested and studied. They have been shown to possess biocompatibility, mucoadhesive properties, and the capacity to modulate epithelial tight junctions [18].

Chitosan-coated liposomes have been demonstrated to improve precorneal retention as well as corneal permeability. Tan et al. showed that the bioadhesive chitosan-coated liposomes containing timolol maleate were more efficient for eye delivery than uncoated liposomes and were also instilled less frequently [88]. In choroidal neovascularization, the chitosan-coated liposomes (CCL) with triamcinolone acetonide had a very high positive surface charge (+41.1 mV) and had a long ocular residence time with good drug diffusion across the corneal mucosal barrier into the vitreous [57].

This was further expanded by Li et al. with a chitosan-coated liposome imparted with the drug triamcinolone acetonide (TA-CHL), which showed high encapsulation efficiency, improved physical stability, and sustained drug release without a high level of cytotoxicity compared to uncoated liposomes [89].

Another strategy to enhance ocular retention of cationic liposomes was reported by Mirkani et al., who developed cationic liposomes coated with methacrylated hyaluronic acid (HAMA), a functionalized hyaluronic acid derivative capable of forming stronger interactions with mucin through both electrostatic and covalent mechanisms.

The uncoated cationic liposomes exhibited a particle size of approximately 170 nm, a PDI of 0.22, and a strongly positive zeta potential. Surface functionalization with HAMA increased the particle size to approximately 285 nm, confirming a successful coating of the liposomal surface.

The HAMA-coated liposomes were designed to utilize a dual muco-adhesion mechanism. First, the positively charged liposomal surface promoted electrostatic interactions with the negatively charged mucin layer covering the ocular surface. Second, methacrylate groups present on HAMA enabled covalent thiol–Michael addition reactions with thiol groups of mucin glycoproteins, resulting in stronger and more prolonged mucosal attachment. Upon exposure to mucin, the liposomes exhibited a substantial increase in particle size (approximately 74%), together with a shift in surface charge toward negative values, indicating strong association with the mucin network.

The mucoadhesive performance of the HAMA-coated liposomes was evaluated against uncoated cationic liposomes and other non-functionalized controls. Ex vivo studies using bovine conjunctival tissue in simulated tear fluid demonstrated significantly greater fluorescence retention for the HAMA-coated formulation, confirming enhanced ocular-surface residence. Drug encapsulation efficiency remained high (approximately 80–86%), while the HAMA coating provided sustained release of bevacizumab, with about 84% of the drug released over 48 h compared with the rapid release of nearly 100% of the free drug within 1 h. These findings demonstrated that HAMA surface functionalization can significantly enhance precorneal retention, prolong drug residence time, and provide controlled drug release, making it a promising strategy for improving ocular bioavailability [90].

The HA-modified liposomes have also been shown to have a longer residence time at the ocular surface. Using confocal microscopy on rabbit eyes, Lin et al. demonstrated that vesicles coated with HA had significantly longer precorneal contact times than uncoated liposomes. Lin et al. also investigated HA-coated lipid-polymer hybrid nanoparticles to deliver moxifloxacin hydrochloride, resulting in better penetration into the eye and greater therapeutic effect [91].

The combination of liposomes that have polymeric matrices with mucoadhesive properties has given especially promising results in hybrid systems. Xu et al. were able to formulate fluconazole into a hyaluronic acid (HA) hydrogel with sustained aqueous humor drug levels above the minimum inhibitory concentration (MIC) for 24 h and reduce the drug’s dosing frequency from 3–4 times daily to once daily while maintaining the patient’s adherence to the treatment regimen [92].

Liposomes coated with a thermosensitive Pluronic hydrogel, administered independently, showed a 12-fold longer residence time in the cornea (60 min) as compared to the liposomes (5 min) in Sprague–Dawley rats [93]. Overall, these hybrid techniques illustrate the combined advantages of surface engineering and polymeric retention strategies. This is further extended by novel biomaterial coatings like that of silk fibroin-coated liposomes, which exhibited increased colloidal stability, corneal muco-adhesion and prolonged drug release compared to uncoated liposomes, indicating that the surface modification of biopolymers is an emerging field for the development of next-generation mucoadhesive ocular drug delivery systems [94].

Khan et al. [90] also showed that surface modification is a good approach to improving ocular drug delivery by increasing the permeation of the cornea with HA- and HAMA-coated cationic liposomes. The uncoated liposomes (170 ±4 nm, ζ potential +51 ± 4 mV) were optimally surface-engineered to provide HA- and HAMA-coated systems with a size of 293 ± 8 nm and 285 ± 6 nm and low zeta potential of +16 ± 2 mV and +24 ± 3 mV, respectively.

This surface modification resulted in a marked improvement in corneal drug permeation. Ex vivo permeation studies demonstrated that cumulative drug permeation increased from approximately 35% for uncoated liposomes to nearly 65% and 70% for HA-coated and HAMA-coated liposomes, respectively, representing approximately a twofold enhancement in drug transport across the cornea.

This is due to increased muco-adhesion and extended precorneal residence that is facilitated by the high interaction with mucin (shift of ζ-potential to −38 mV and increase in size of approximately 74 percent). Particularly, the increased efficacy of HAMA-coated liposomes underscores the value-added effect of covalent interaction in addition to electrostatic binding, which leads to a longer retention time on the ocular surface with better bioavailability [90].

Tan et al. [88] showed that the use of chitosan as a bioadhesive polymer coating of the liposomal drug delivery systems is a useful approach to improve corneal permeability and ocular bioavailability. The fabricated chitosan-coated liposomes (TM-CHL) had desirable physicochemical characteristics, such as a particle size of 150.7 nm and entrapment efficiency of 75.83 ±1.61%, aiding in the effective encapsulation of drugs.

The bioadhesive property of chitosan significantly enhanced corneal transport. The apparent permeability coefficient (Papp) increased by 3.18-fold as compared to conventional eye drops, reaching 1.27 ± 0.17 × 10^−5^ cm/s versus 0.40 ± 0.05 × 10^−5^ cm/s. A further improvement was seen in steady-state flux, which increased from 2.44 ± 0.23 to 5.97 ± 0.09 µg/s/cm^2^. Additionally, TM-CHL was found to have longer precorneal retention and enhanced pharmacokinetic behavior, as it increased the AUC by 3.9 times and achieved a Cmax (257.43 ± 8.94 µg/mL) that was significantly higher, indicating a much greater bioavailability.

These are attributed to the mucoadhesive bond of chitosan with corneal mucin and the capacity of chitosan to temporarily open epithelial tight junctions, which promote long-term drug residence and increased absorption [88].

Conclusion: Mucoadhesive coatings, particularly covalently anchored formulations such as HAMA, provide the second most reproducible enhancement in this review, nearly doubling cumulative corneal permeation relative to uncoated liposomes across nine independent studies [18,88,89,90,91,92,93,94]. The consistent hierarchy of covalent > electrostatic > uncoated mucin binding indicates that reaction chemistry, not merely surface charge, is the dominant determinant of mucoadhesive performance.

### 3.5. Bile-Salt Incorporation

It has been shown that the incorporation of bile salts into liposomal formulation is a successful method that enhances the permeability of the cornea and the bioavailability of the drug in the eye. This is achieved by making the vesicles flexible, increasing transmembrane diffusion and temporarily opening tight junctions between epithelial cells to allow paracellular transport.

Tacrolimus-loaded liposomes containing sodium taurocholate (STC), sodium deoxycholate (SDC) or sodium glycocholate (SGC) were prepared by the thin-film dispersion method with the production of nanosized vesicles (92.8–98.1 nm) with high entrapment efficiencies (92.11–96.53%). The zeta potential of the liposomes was highly negative (–21.9 ± 3.1 mV) in comparison to that of the conventional cholesterol liposomes (–1.2 ± 0.3 mV) due to the incorporation of bile salts that prevented aggregation, thus improving the stability of the vesicles.

In addition, tampering with the particle size from ~80 nm to ~90 nm by ultrasonication did not affect EE, as tacrolimus highly binds to the lipid bilayer. All bile-salt liposomes resulted in a sustained drug release profile with less than 5% of tacrolimus released after 24 h, which suggests a longer ocular residence time.

The ex vivo transcorneal-permeation studies showed a significant improvement in penetration after the addition of bile salts. STC-liposomes showed the highest apparent permeability coefficient (Papp 36.24 ± 3.51 × 10^−8^ cm/s), representing a 4.5-fold increase over conventional cholesterol liposomes (8.00 ± 2.05 × 10^−8^ cm/s), while SDC- and SGC-liposomes showed 3.7-fold increases with Papp values of 29.50 ± 5.78 × 10^−8^ cm/s and 29.73 ± 4.03 × 10^−8^ cm/s, respectively. This was confirmed by confocal microscopy, which demonstrated that bile-salt liposomes diffused more rapidly and deeply into the cornea, with strong fluorescence remaining across the epithelium, stroma, and endothelium for up to 60 min, whereas conventional liposomes had weak fluorescence by 60 min.

The enhancement of the binding effect of bile salts on the liposomal membrane was also found to increase the deformability and fluidity of the membrane, facilitating the diffusion of liposomes across corneal layers, thereby improving the corneal permeability of liposomes. The high deformability and fluidity of the liposomal membrane also allowed liposomes to temporarily disrupt tight junctions, thus increasing the paracellular permeability of the cornea. Sodium taurocholate and sodium glycocholate were found to be well tolerated by the eye with corneal hydration of 78.23 ± 0.94% and within a normal physiological range (<83%), respectively, while sodium deoxycholate exhibited more cytotoxic activity and corneal damage because of its stronger surfactant activity [95].

The permeation enhancers used in liposomal systems (bilosomes) are bile acids and bile salts, which have been incorporated into bilosomes to improve the ocular bioavailability and corneal permeation by inducing membrane fluidity, enhancing vesicle deformability, transiently loosening the tight junctions in the epithelial barrier, and increasing the transcellular and paracellular transport across the ocular barriers.

Bile salts containing liposomes have better stability than conventional liposomes due to the fluidization of the lipid bilayer by bile salts and their ability to stabilize vesicles from disruption. These properties enhance drug transport across the membrane and improve the ocular retention and stability of the liposomes. An increase in systemic delivery of insulin from 1% to 5.5% of sodium glycocholate (NaGC) (1%) co-administered with insulin was confirmed by ocular permeation studies in rabbits that showed bile salts to be very effective for enhancing the ocular absorption of insulin.

Likewise, the permeation of β-blockers across isolated rabbit corneas was increased by the addition of bile salts; 0.05% taurodeoxycholate (TDC) increased the permeation of atenolol by 5.8-fold, while 0.05% deoxycholate (DC) and ursodeoxycholate (UDC) increased the permeation of timolol by 5.2-fold and 2.1-fold, respectively. The effect of bile salts on the bioavailability in the eyes is a result of the breakdown of the lipid organization of the corneal epithelium, the increase in permeability and the transient opening of tight junctions, which allow greater penetration of the drug through the corneal tissues.

Bilosomes have been developed using bile acids and bile salts as permeation enhancers to enhance the bioavailability of the drug in the eye and the permeation of the drug through the cornea by increasing membrane fluidity, vesicle deformability, transiently loosening epithelial tight junctions and promoting the transcellular and paracellular transport across the barriers of the eye. Bile salts in liposomes are more stable than conventional liposomes, as they first fluidize the lipid bilayer and then stabilize vesicles from membrane disruption, which enhances transmembrane drug transport and ocular retention.

Ocular permeation studies were performed in rabbits and showed that the co-administration of sodium glycocholate (NaGC, 1%) raised insulin delivery to systemic circulation by 5.5% from 1%. This means that the co-administration of bile salts can increase the absorption of insulin in the eye significantly. Likewise, when compared to permeation across isolated rabbit corneas, 0.05% taurodeoxycholate (TDC) stimulated corneal permeation of atenolol by 5.8-fold, 0.05% deoxycholate (DC) stimulated timolol permeation by 5.2-fold and 0.05% Urso deoxycholate (UDC) stimulated it by 2.1-fold.

The mechanism through which bile salts enhance the ocular bioavailability includes the breakdown of the lipid organization of the corneal epithelium, the increased permeability of the membrane, and transiently permeabilizing the tight junctions, which allows for greater penetration of the corneal tissues. The drug-transport-facilitating action of bile salts is complemented by the sustained release and protective action of liposomal vesicles in bile-salt-containing liposomes (bilosomes).

These systems enable better diffusion of drugs through biological membranes and the stability and long-term storage of vesicles. The permeability of salmon calcitonin increased 10.8-fold with the use of proliposomes containing sodium taurodeoxycholate (NaTDC) at 0.1% in permeation studies. The oral bioavailability of fenofibrate was improved by 5.1-fold when formulated into bilosomes containing NaDC (SDS) compared to what was obtained for micronized formulations. For cyclosporine A-loaded NaDC liposomes, the relative bioavailability was 120.3%, compared with 98.6% for conventional cholesterol liposomes. Pavlovic et al. provide a comprehensive mechanistic framework for understanding bile-acid-mediated permeation enhancement.

This study identified six distinct modes of action relevant to biological membrane interactions that include increased solubilization of lipophilic drugs, membrane fluidity enhancement at submicellar concentrations through partitioning into the phospholipid bilayer, reversible tight-junction opening via calcium ion chelation, the formation of reverse micelles, creating transient aqueous transmembrane channels, the inhibition of P-glycoprotein efflux, and direct membranolytic activity at concentrations exceeding the critical micellar concentration (CMC).

The CMC values of naturally occurring bile acids range from 2 to 20 mM in aqueous media, with hydrophobicity being the primary determinant of self-assembly behavior and membrane interaction capacity—increasing in the order UDCA < CA < CDCA < DCA < LCA. For ocular formulation design, bile salts at submicellar concentrations increase membrane fluidity and permeability without causing irreversible damage, whereas concentrations above the CMC risk membranolytic effects.

This concentration-dependent duality underscores the importance of precise bile-salt loading ratios in liposomal ocular systems. The facial amphiphilicity of bile acid molecules characterized by a concave hydrophilic alpha-surface bearing 1–3 hydroxyl groups and a convex hydrophobic beta-surface distinguishes them structurally from conventional surfactants and confers their unique capacity to simultaneously solubilize both polar and non-polar drug molecules within primary and secondary micellar aggregates, respectively [96].

Of direct translational relevance to ocular delivery, Saettone et al. demonstrated that taurodeoxycholate (TDCA) at a concentration of 0.05% produced the most pronounced permeation-enhancing effect across isolated rabbit cornea for a panel of beta-blocking agents, increasing the permeation of the hydrophilic drugs atenolol and timolol more efficiently than the lipophilic drugs levobunolol and betaxolol, without elevating corneal hydration levels beyond the safety threshold and without any in vivo irritant activity at this concentration [97]. This finding directly informs the selection of bile-salt identity and concentration in the formulations discussed in this section. Collectively, these findings establish bile-salt incorporation as a mechanistically distinct liposomal permeation-enhancement strategy capable of significantly improving ocular and mucosal drug absorption through enhanced membrane interaction and trans-barrier transport [98].

Conclusion: Bile-salt incorporation produces the largest single fold-enhancements in corneal permeability reported in this review (up to 4.5-fold [95], driven by the dual mechanism of membrane fluidization and transient tight-junction opening). Because these same mechanisms become membranolytic above the critical micellar concentration, bile-salt selection and concentration must be individually optimized: sodium taurocholate and sodium glycocholate offer the most favorable efficacy-tolerability balance among the salts reviewed, whereas sodium deoxycholate is comparatively more cytotoxic [95].

### 3.6. Dendrimer-Assisted Surface Engineering

Dendrimers are highly branched, well-defined polymeric structures that have been explored as surface modifiers to enhance the performance of liposomal ocular drug delivery systems. Lai et al. developed compound liposomes co-encapsulating berberine hydrochloride (BBH) and chrysophanol (CHR) and coated them with poly(amidoamine) (PAMAM) dendrimers. These were identified as third-generation (G3.0) dendrimers, as confirmed by FITC labeling and ^1^H-NMR spectroscopy. The PAMAM coating increased the encapsulation efficiency of both drugs, which was attributed to electrostatic interactions between the amine-rich dendrimer surface and the drug molecules. In addition, the coating altered the zeta potential of the vesicles, resulting in a surface charge more favorable for corneal interaction and improved ocular delivery performance [99,100,101].

Biologically, PAMAM-coated liposomes (P-CBLs) demonstrated superior cellular uptake in vitro and greater corneal penetration in vivo compared to uncoated formulations. Ocular microdialysis revealed higher aqueous humor concentrations and prolonged residence times for BBH in P-CBL-treated animals, along with elevated C_max_ values [102]. At the retinal level, P-CBLs provided superior protection against light-induced retinal damage, preserved retinal structure and function, and reduced intracellular oxidative stress in vitro [103,104,105,106]. Repeated administration produced no observable ocular irritation or structural damage, supporting the safety of the dendrimer-coated platform. These findings position PAMAM G3.0-coated liposomes as a potentially transformative delivery mode for AMD and other posterior-segment diseases [99].

Conclusion: PAMAM dendrimer coating is the least independently replicated strategy in this review, with the available evidence derived from a single formulation system (refs. [99,100,101,102,103,104,105,106]). While the reported combination of enhanced corneal penetration and retinal antioxidant protection is promising, broader validation across additional drugs, dendrimer generations, and independent laboratories is needed before its reproducibility can be assessed on par with cationic or mucoadhesive strategies.

### 3.7. Ligand-Targeted Surface Modification

Beyond passive retention strategies, liposomal surfaces can be actively functionalized with targeting ligands. These targeting ligands include antibodies, peptides, and receptor-specific agonists. These receptor-specific agonists convert non-selective vesicle distribution into receptor-mediated, cell-specific drug delivery at the ocular target site.

Li et al. established that surface functionalization of nanocarriers with targeting ligands enables selective receptor interaction at specific ocular tissues, concentrating drug at the site of action while limiting systemic distribution. It is critically important to recognize that conventional topical delivery reaches as little as <5% of the applied dose at the retina [107]. Juliana et al. [107] demonstrated that liposomes conjugated with a peptide agonist for alpha-2 adrenergic receptors achieved improved ocular drug delivery and significant IOP reduction in a glaucoma model compared to unconjugated formulations.

This was a significant advancement, given that the equivalent free drug brimonidine, despite achieving IOP reductions of 4 to 6 mmHg clinically, is limited by rapid precorneal clearance and ocular allergy rates of 10 to 30% with conventional topical dosing [108].

The pharmacodynamic advantage of surface-modified liposomes in this indication is further evidenced by positively charged acetazolamide-loaded liposomes achieving a peak IOP reduction of 7.8 mm Hg at 3 h and sustained over 8 h in rabbit eyes. This markedly exceeded free drug treatment duration with receptor-specific ligand conjugation expected to amplify this further by directing vesicles precisely to trabecular and RGC targets rather than relying on non-specific corneal contact [109].

The multivalency of the liposomal surface enables the simultaneous engagement of multiple receptor copies per vesicle and confers binding avidity and cellular uptake efficiency unachievable by monomeric drug-receptor interaction alone. Notably, brimonidine delivered at 1 mg/kg/day via an osmotic pump significantly reduced RGC loss from 33% to 15% in a chronic ocular hypertensive rat model, underscoring the neuroprotective ceiling that targeted liposomal delivery of this agent could approach if adequate posterior-segment concentrations are achieved [107,108].

The evidence for receptor-mediated liposomal targeting at retinal tissues is further substantiated by transferrin receptor-targeted systems. Managit et al. developed transferrin-conjugated ganciclovir-loaded liposomes (Tf-GCV-LPs) with particle sizes of <100 nm that were internalized by human retinal pigment epithelial cells (ARPE-19) specifically via transferrin receptor-mediated endocytosis. This has shown a significant elevation of intracellular ganciclovir concentrations at 24 h and produced the highest inhibitory activity against CMV glycoprotein B of all tested formulations. This demonstrated that receptor conjugation redirects liposomal uptake from non-specific surface contact to active, cell-targeted internalization at the retinal level [110].

More recently, Luo et al. [111] demonstrated that Tet1 peptide-functionalized liposomes selectively targeted retinal ganglion cells (RGCs) in vivo. These liposomes enhanced penetration across the inner limiting membrane. They also increased RGC-specific drug uptake while minimizing off-target glial accumulation. This targeted delivery significantly promoted RGC survival. It further supported neurite outgrowth and axonal regeneration in an optic nerve crush model. Importantly, no retinal inflammation was observed. This is the first demonstration of peptide-targeted liposomal delivery achieving both neuroprotective and regenerative outcomes at the RGC level [111].

A wide variety of penetration enhancers have been incorporated into liposomal or nanoparticulate systems for ocular delivery to increase the bioavailability of the encapsulated drug in the posterior segment of the eye, to avoid precorneal clearance and to enhance penetration across the tight corneal junctions. Within these, peptides containing cell-penetrating sequences (CPPs) like transactivator of transcription (TAT) peptide, penetratin, and arginine-glycine-aspartic acid (RGD)-modified peptides have been found to be most effective in delivering to the retina when linked to liposomes or polymeric nanocarriers. The TAT peptide promotes cellular uptake through electrostatic interactions with the negatively charged cell membrane, triggering internalization via macropinocytosis and clathrin-mediated endocytosis. In contrast, unconjugated endostatin formulations demonstrated poor retinal uptake, whereas TAT-conjugated formulations significantly enhanced retinal delivery and accumulation.

Although the unconjugated endostatin formulations were poorly taken up into the retina, the TAT-conjugated ones exhibited efficient topical retinal distribution. TAT-endostatin-RGD formulations yielded ~14-fold higher retinal concentrations of the drug (9.42 ± 0.77 ng/mg) compared to the unconjugated drug (0.68 ± 0.10 ng/mg) and sustained the presence of the drug in the retina for ~8–11 h in retinal delivery studies.

Likewise, the fluorescence intensity for retina-choroid tissues was ~11-fold higher for the modified nanoparticles compared with non-modified nanoparticles. Eye-to-retina transport was raised from 1.23 ± 0.56% to 3.66 ± 0.67%, respectively, showing a marked improvement in the bioavailability and targeted accumulation in the retina of dual-modified nanoparticles.

Penetratin is another strongly investigated cell-penetrating peptide (CPP), which is derived from homeodomain protein Antennapedia and which is used to achieve improved permeation in the eye by transcorneal and conjunctival-scleral pathways. The fluorescence intensity was found to be significant within 10 min after topical instillation, reaching the maximum level at 30 min in 8 h, and holding at detectable levels up to 12–24 h and suggesting a long retention time in the retina.

Poly(amidoamine) (PAMAM) dendrimer nanocarriers modified with penetratin were found to penetrate throughout the layers of the retina, such as the retinal pigment epithelium, photoreceptor layer, ganglion cell layer and outer plexiform layer. The modification of nanocarriers with penetratin also resulted in an increase in the intensity of fluorescence in the retina, which was ~3-fold higher than that of the non-modified nanocarriers, further evidence of the enhanced penetration and accumulation in the retina.

Liposomal surface modifiers including mucoadhesive polymers, such as chitosan and carboxymethyl chitosan (CMCS), have also been used for extending the precorneal residence time and ocular duration of the drug. The posterior-segment signal intensity of chitosan-coated liposomes was higher than that of non-coated liposomes. The residence time of drugs was higher than that of non-coated liposomes, in part because the drug remained in the eye for up to 12 h, compared to 10 h for non-coated liposomes, probably due to the increased muco-adhesion and decreased tear washout. Likewise, the drug concentrations in the retina and choroid of the CMCS coated drug delivery nanocomposites were 2.5 times higher than those of the non-coated drug delivery systems.

A detectable amount of the drug was present for up to 3 h after administration, whereas eye drops showed drug concentration below the detection limit. This enhancement was attributed to the long residence time of these nanocarriers in the conjunctival sclera, as well as to the clathrin-mediated endocytosis of the particles. Benzalkonium chloride (BAC), however, is a surfactant that is widely used as a preservative for ophthalmic preparations, but which showed only moderate improvements in retinal bioavailability when administered as nanoformulations.

The main mechanisms of BAC’s effect on corneal permeation include disruption of the tight junctions of the corneal epithelium and an increase in the fluidity of the membranes. Its effect on delivery to the posterior segment was significantly lower than those of the CPP-or chitosan-based systems. Overall, these results confirm that the improvement in the ocular bioavailability is the main mechanism of action of the CPP-mediated liposomal and nanoparticulate system, while the chitosan-based coating system enhances precorneal retention and extended ocular residence, thus complementing each other in the optimization of topical ocular drug delivery [112].

Conclusion: Ligand-targeted and cell-penetrating-peptide systems achieve the largest reported posterior-segment enhancements of any strategy in this review, including a 14-fold increase in retinal drug concentration with TAT-RGD conjugation [112]. Because this effect is governed by the specific ligand-receptor pairing rather than a shared physicochemical property, magnitude is not directly comparable across ligands, and reproducibility should be assessed strategy by strategy rather than assumed to generalize across the class.

### 3.8. Ultra Deformable/Edge-Activator-Modified Liposomes (Transferosomes)

Transferosomes (TFS) represent a structurally evolved subclass of liposomes that are incorporated with an edge activator. Edge activators are a single-chain surfactant such as Tween 80, Span 80, or a bile acid such as sodium cholate incorporated into the phospholipid bilayer. The incorporation of edge activators confers extreme membrane deformability without disrupting vesicular integrity.

This bilayer-level modification reduces the elastic modulus of the phospholipid backbone, enabling vesicles to undergo reversible deformation and squeeze through intercellular pores smaller than their own diameter. This is a property that rigid conventional liposomes fundamentally cannot replicate. A further driving force is trans-pore hydrotaxis, whereby the high surface hydrophilicity of TFS promotes osmotic gradient-driven migration from lipophilic epithelial tissue toward the richly perfused hydrophilic deeper tissue layers, a mechanism with direct implications for both corneal and conjunctival drug penetration.

Barbalho et al. [109] developed curcumin-loaded TFS using Phospholipon 90G and 10% (*w*/*w*) Tween 80 as the edge activator by thin-film hydration method, producing vesicles with a particle size of 143 ± 1.16 nm, PDI of 0.09 ± 0.02, zeta potential of −14 ± 0.69 mV, and entrapment efficiency of >99.96%. The increase in entrapment efficiency was due to the hydrophobic nature of curcumin driving preferential localization within the lipid bilayer. In vitro release demonstrated a favorable triphasic release profile that includes an initial minimal burst release of <2% within the first 15 min, followed by a negligible lag phase until 2 h, with a final sustained release phase (the small burst release) being particularly advantageous for ocular delivery, as it minimizes reflex tearing and precorneal drainage that would otherwise rapidly clear conventional eye drops.

Corneal biocompatibility was confirmed by the bovine corneal opacity and permeability (BCOP) assay, with TFS receiving a cumulative irritation score of 0.0. This led to classifying them as practically non-irritating, with no fluorescein staining, corneal opacity, or epithelial wrinkling being observed, in stark contrast to positive controls, 5% benzalkonium chloride (BAK) (score 6.2), 0.1N NaOH (score 6.5), and acetone (score 8.0).

Ex vivo penetration studies in a porcine eye model revealed that TFS achieved significantly superior drug penetration across all three evaluated ocular tissues relative to the conventional oily control solution. In the cornea, total curcumin penetration from TFS was evaluated by digital image analysis using ImageJ 1.54 series software. The amount of curcumin penetration was determined semi-quantitatively by analyzing the mean gray value per pixel (MGV/px) in the image, and it was found to be8 ± 0.6 (MGV/px), which was more than 2-fold greater than the oily control (3 ± 0.3 MGV/px). The mean penetration depth was 57 ± 7.5 μm, representing a 1.6-fold improvement over the control (36 ± 9.2 um).

Confocal microscopy also confirmed penetration beyond the epithelial layers into the hydrophilic stroma. The penetration-enhancing effect was even more pronounced in conjunctival tissues. In the bulbar conjunctiva, TFS achieved a 4-fold greater total drug penetration (12 ± 0.8 MGV/px versus 3 ± 0.3 MGV/px) with a mean penetration depth of 70 ± 15.1 μm. This was approximately 14-fold greater than the oily control (5 ± 3.8 um).

In the tarsal conjunctiva, the total penetration was 2.5-fold higher (16 ± 2.9 MGV/px versus 3 ± 0.8 MGV/px) with a mean depth of 83 ± 14. The tissue-dependent hierarchy of TFS penetration was the greatest in the tarsal conjunctiva, followed by the bulbar conjunctiva and cornea, respectively. This directly reflects the 230-fold larger intercellular spaces of the conjunctival epithelium relative to the cornea, confirming that the osmotic gradient-driven deformability of TFS is most effectively exploited in tissues with a more permissive intercellular architecture.

Critically, the authors identified the conjunctival-scleral pathway as a potentially significant complementary route for TFS distribution into the iris, ciliary body, uvea, and choroid/retinal pigment epithelium. This study further confirms the therapeutic relevance of this platform beyond anterior segment targets to posterior-segment diseases where non-invasive access has historically been severely limited [109].

The clinical need for enhanced ocular antifungal delivery is underscored by the shortcomings of natamycin 5% (*w*/*v*) suspension (Natacyn^®^), which remains the only FDA-approved topical antifungal therapy despite its poor ocular bioavailability and frequent dosing requirements.

Natacyn, which achieves ocular bioavailability of <5%, owing to rapid precorneal clearance and nasolacrimal drainage, requires dosing as frequently as 1 drop every 1–2 h for the first 3–4 days, followed by 6–8 times daily for an additional 14–21 days. This mode of topical therapy can resolve fungal infections in only 7.6% of patients, with 92.4% requiring surgical intervention. Vesicular surface-engineered systems have consistently demonstrated superiority over this benchmark. Natamycin-loaded transferosomes achieved approximately 9-fold improved corneal permeability over drug suspension alone, which increased further to approximately 16-fold when the transferosomes were incorporated into an ion-sensitive in situ gellan gum gel. Natamycin bilosomes loaded into a gellan gum in situ hydrogel achieved 6- to 9-fold greater transcorneal flux compared to Natacyn and produced significantly higher natamycin concentrations in ocular tissues at 6 h post-administration.

An MTT assay and rabbit corneal histology confirmed the non-toxicity of the gellan gum carrier. Glycerosome-based natamycin eye drops demonstrated an encapsulation efficiency of 80.84% versus 59.5% for conventional liposomes. The ex vivo corneal penetration was approximately 80% versus 75% for conventional liposomes and 20% for the marketed natamycin eye drops, which was attributed to glycerol-enhanced membrane deformability, improving penetration across ocular barriers [113].

Taken together, these findings confirm that the surface and structural modification of liposomal and vesicular carriers, whether through edge activator incorporation, bile-salt integration, or glycerol-mediated deformation, consistently converts a drug with poor inherent corneal penetration into a therapeutically viable antifungal platform, addressing both the bioavailability deficit and the unacceptably high dosing burden of the current standard of care.

Conclusion: Edge-activator-modified liposomes reproducibly favor conjunctival-scleral over corneal delivery, with a consistent tissue-penetration hierarchy (tarsal conjunctiva > bulbar conjunctiva > cornea) replicated across the formulations tested [109]. This tissue selectivity, combined with a favorable irritation profile [109], positions transferosomes as a complementary rather than a primary corneal-permeation strategy relative to cationic or mucoadhesive approaches.

Table 5 summarizes the classic examples of surface-modified liposomal ophthalmic formulations mentioned above and Table 6 provides comparative analysis of several surface-modified strategies of liposomal ophthalmic formulation.

#### 3.8.1. Integrated Mechanistic Discussion of Surface-Modification Strategies

Across the seven surface-modification strategies reviewed, the degree of benefit tracks closely with how many independent barrier-crossing mechanisms a formulation engages at once, and this pattern also predicts how reproducible each strategy’s results are across independent laboratories.

#### 3.8.2. Best-Performing and Most Reproducible Surface-Modification Strategies

Cationic surface-charge modification and mucoadhesive polymer coating show the most reproducible benefit: both improve permeation and retention irrespective of the specific drug tested, and both are supported by the largest independent evidence base in this review (~10 and ~9 studies, respectively) [14,18,74,75,76,77,78,79,80,81,82,83,88,89,90,91,92,93,94]. Bile-salt incorporation produces the single largest fold-changes in corneal Papp (up to 4.5-fold, ref. [95]), but this advantage is concentration-dependent and can reverse into cytotoxicity, which lowers its practical reproducibility relative to the two strategies above.

#### 3.8.3. Mechanistic Basis for the Performance Differences Across Various Surface-Modification Strategies

Strategies that engage two independent barrier-crossing mechanisms simultaneously outperform single-mechanism approaches. HAMA-coated cationic liposomes combine electrostatic adsorption with covalent thiol–Michael mucin binding and outperform HA-coated (electrostatic-only) and uncoated liposomes in a fixed dose-ordering [90]. Bile salts similarly act through both membrane fluidization and reversible tight-junction opening [96], which explains why their fold-enhancements exceed single-mechanism coatings. PEGylation, by contrast, relies on one passive mechanism (steric shielding) and is measurably weaker unless paired with a second, active mucoadhesive mechanism [85]. Ligand-targeted and dendrimer systems achieve the largest posterior-segment gains because they substitute passive diffusion for active, receptor- or charge-driven uptake—but this specificity also ties their effect size to the individual ligand-receptor pair, rather than a generalizable physicochemical property, which is why cross-study reproducibility is lower for this class.

#### 3.8.4. Reproducibility of Reported Results

The effect direction (i.e., that the modification improves permeation/retention relative to unmodified liposomes) is reproducible across all seven strategies. The effect magnitude is reproducible only for cationic charge and mucoadhesive coating, with independent laboratories using different drugs converging on comparable fold-ranges (2- to 5-fold). For bile salts, dendrimers, and ligand-targeted systems, the magnitude varies substantially with a formulation-specific variable (bile-salt identity/concentration, dendrimer generation, or ligand-receptor pair), so numerical comparisons between studies should be treated as indicative rather than directly generalizable (see Table 7).

No single surface-modification strategy for liposomal formulations for ophthalmic use is universally optimal. Cationic and PEGylated liposomes currently possess the highest translational readiness. However, the ligand-targeted, dendrimer-modified, and multifunctional hybrid liposomes offer the highest therapeutic sophistication but face greater manufacturing and regulatory challenges. Table 8 shows a comparative analysis of different surface-modification strategies in terms of mechanism of corneal penetration, corneal permeability, ocular residence time, drug-loading, safety and clinical translational status. Table 9 shows the advantages and disadvantages of surface-modification strategies for liposomal ophthalmic formulations. The future clinical success of surface-modified liposomes for ophthalmic use will likely arise from hybrid systems that integrate muco-adhesion, permeability enhancement, and active targeting while maintaining manufacturability and regulatory simplicity.

#### 3.8.5. Mechanism of Drug Transport for Surface-Modified Liposomes

**Figure 4:**
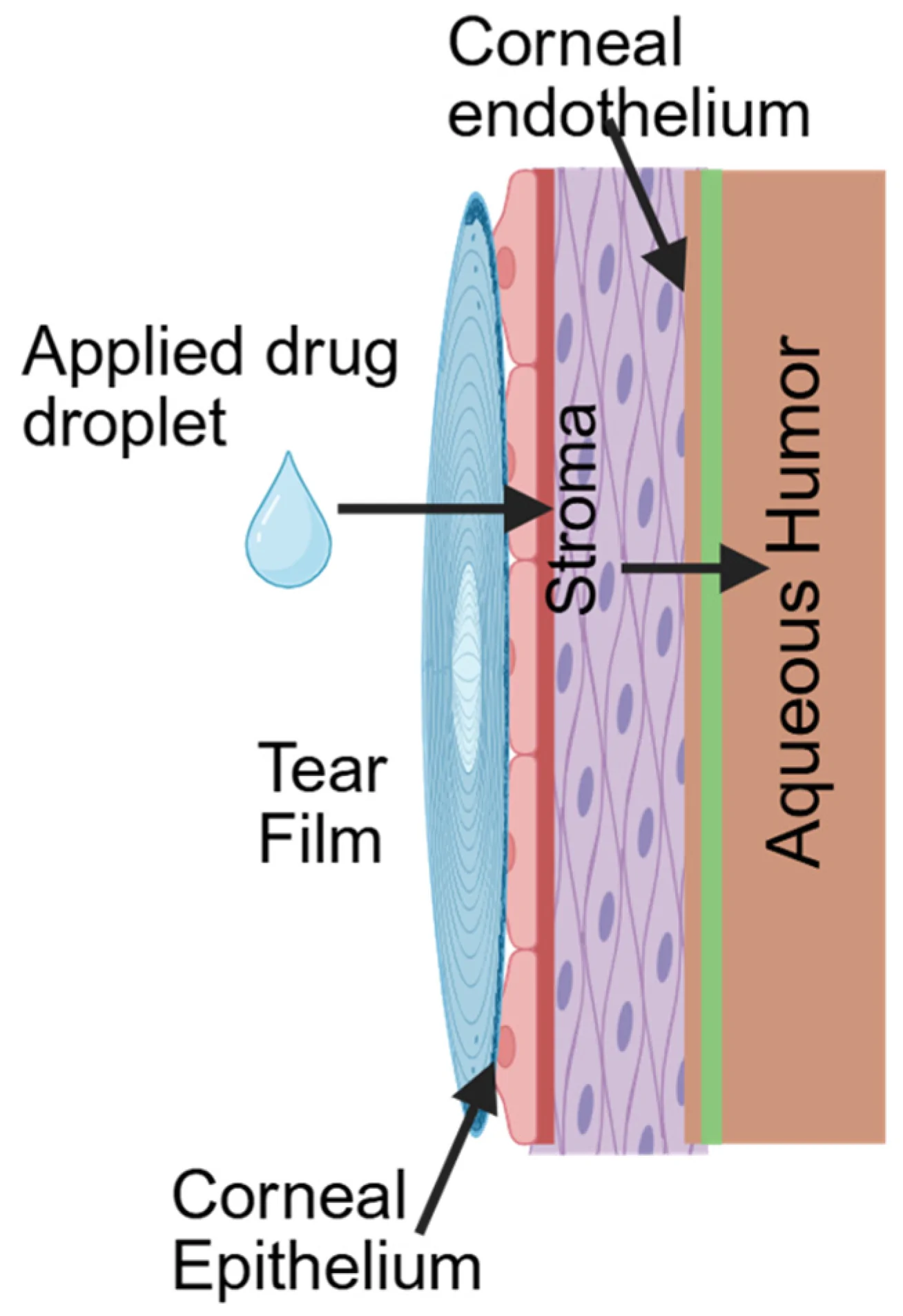
Drug absorption through corneal membrane via transcellular route. Created in BioRender. Moore, E.D. (2026). https://app.biorender.com/illustrations/6a777961de3ceba64f735776 (made using BioRender).

**Figure 5:**
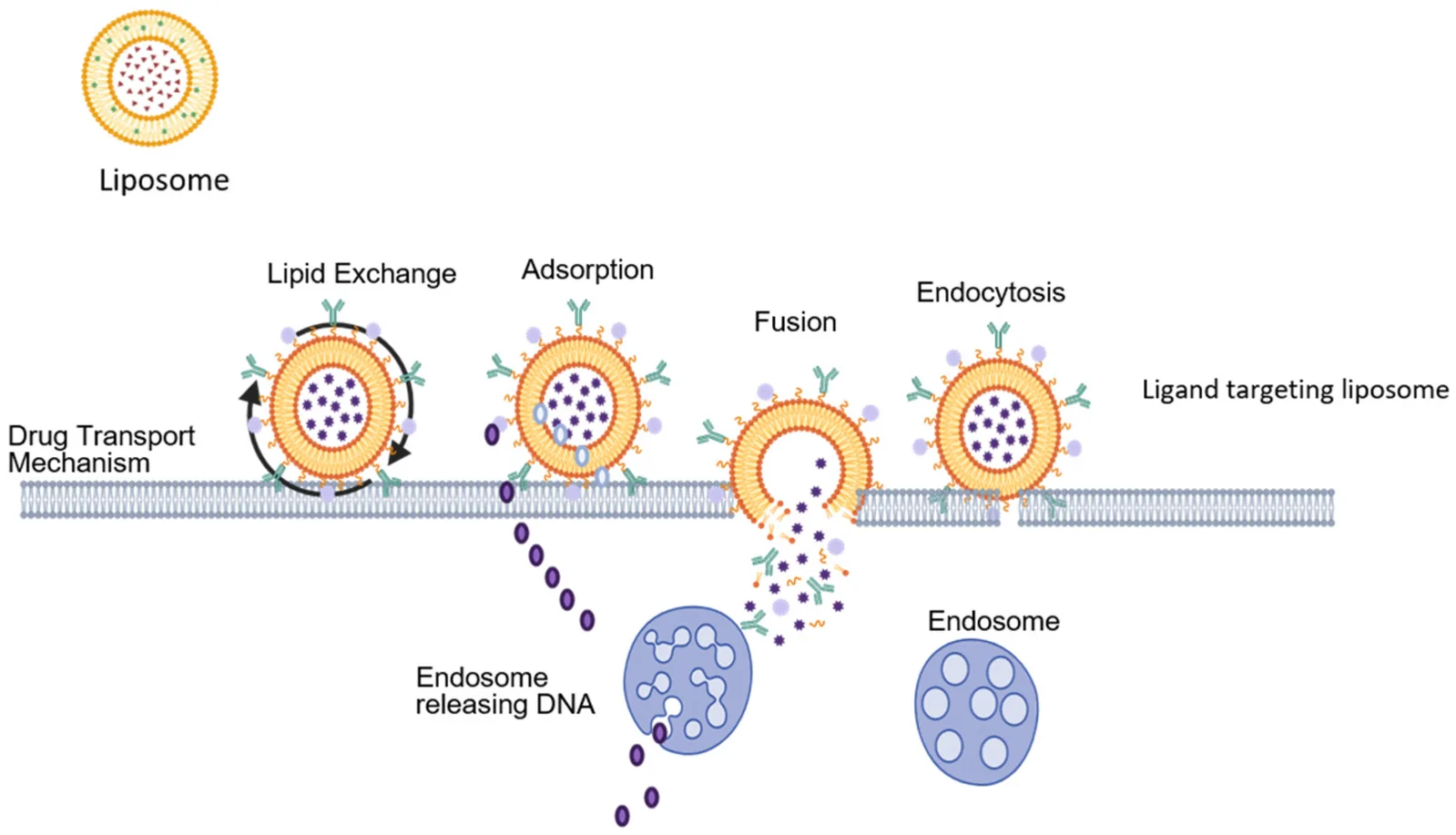
Basic mechanism of drug transport via liposome carrier. Created in BioRender. Moore, E.D. (2026). https://app.biorender.com/illustrations/6a777961de3ceba64f735776 (made using BioRender).

**Figure 6:**
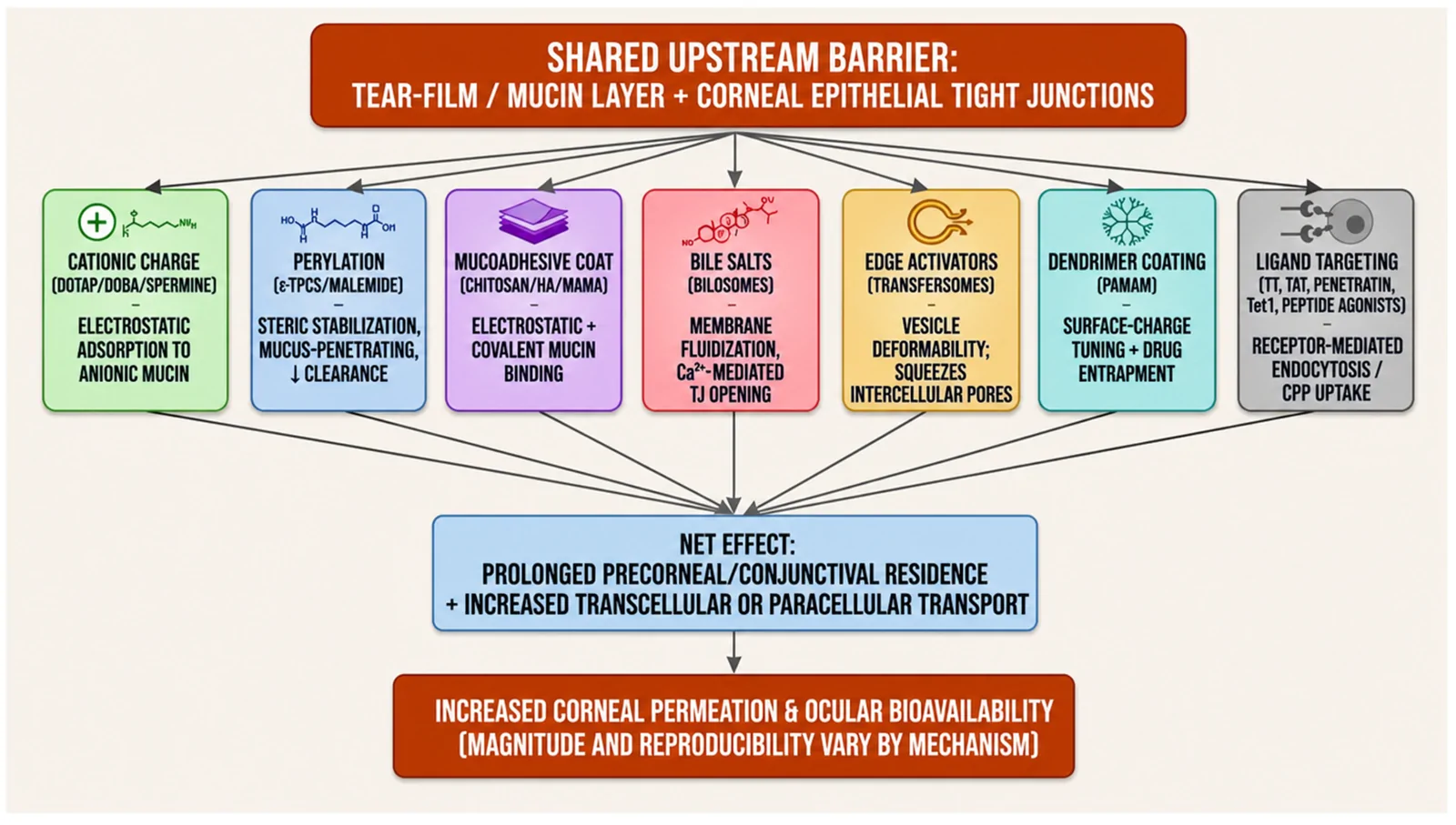
Convergent mechanisms of liposomal surface modification in overcoming ocular barriers. Made using FigureLabs.

The topical delivery of a drug into the cul-de-sac of the eyes occurs via two routes of drug penetration: (1) the non-corneal route (diffusion through the sclera and conjunctiva) and (2) the corneal route via corneal-membrane-reaching ocular tissue (Figure 4 depicts the drug absorption through the corneal membrane via transcellular transport). Transcellular transport through the corneal membrane and stroma presents the major mechanism of the absorption of drugs. Drug transport through the liposome involves a lipid transfer or exchange through adsorption to the cellular membrane, which occurs through weak hydrophobic interaction or electrostatic interaction, as in cationic liposomes and endocytosis by phagocytosis via reticuloendothelial cells (Figure 5 shows the mechanism of drug transport through liposomes). Ligand-targeting liposomes transport drug via receptor-mediated uptake. Figure 6 shows a convergent mechanism of drug transport via surface-modified liposomes in overcoming ocular barriers and improving precorneal residence time and ocular bioavailability.

### 3.9. Clinical Applications and Loading Strategies

Apart from surface modification, the loading protocol of liposomes with drugs can also have a great impact on the encapsulation efficiency, formulation stability, and in vivo performance. Compared to passive loading methods, active loading techniques with the help of pH or ion gradients always result in a higher level of encapsulation of the drugs in the aqueous core of the liposomes.

Fujisawa et al. used the calcium acetate gradient technique for loading the molecules of diclofenac into liposomes with 97% encapsulation efficiency. The resulting formulations allowed a 1.8-fold improvement in the distribution of the drug throughout the retina and choroid as compared to conventional diclofenac eye drops. Further, this method was optimized by a surface modification of the liposomes with polyvinyl alcohol (PVA) to improve the stability and corneal permeability, leading to a significantly higher concentration of the drug in the posterior segment of the eye from the diclofenac liposomes [100]. Active loading was also employed in the preparation of liposomes via the reverse-phase evaporation technique, which produces large unilamellar vesicles with high drug entrapment and sustained release, as demonstrated for fluconazole in a Candida keratitis rabbit model, where superior therapeutic outcomes were achieved relative to conventional eye drops [115].

Surface-modified liposomal systems have demonstrated consistent and reproducible therapeutic superiority over conventional ophthalmic preparations across a broad spectrum of anterior and posterior-segment diseases, with the nature of the advantage varying predictably by indication. In fungal and bacterial keratitis, the primary gain is in corneal drug penetration and precorneal residence. Liposomal formulations of antifungal agents such as voriconazole, flucytosine, and rapamycin, and antibacterials such as ciprofloxacin, uniformly achieve higher transcorneal permeation, more sustained local drug concentrations, and greater microbiological efficacy relative to conventional solutions, suspensions, and even gel-based systems, without added irritation [116,117,118,119,120,121].

In inflammatory and immune-mediated diseases such as dry eye disease, autoimmune uveitis, and macular edema, liposomal delivery offers a dual advantage. Enhanced anterior segment bioavailability reduces the need for frequent dosing. Sustained release helps maintain therapeutic drug levels over clinically relevant time periods.

For example, triamcinolone acetonide-loaded liposomes have shown significant improvement in best-corrected visual acuity and central foveal thickness without increasing intraocular pressure. Similarly, infliximab-loaded liposomes have demonstrated a marked reduction in retinal inflammatory infiltration and tissue damage compared to free biologic formulations in uveoretinitis models [114,122,123,124].

For posterior-segment targets, including diabetic retinopathy and gene therapy applications, liposomal and lipid nanoparticle platforms address the more fundamental challenge of transscleral and intravitreal delivery to tissues that are pharmacokinetically inaccessible to conventional topical formulations. However, with surface modifications like cationic charge, hyaluronic acid grafting improves adequate tissue penetration, and in the case of nucleic acid payloads such as IL-10 plasmids, measurable transgene expression at the corneal epithelium could be achieved [125,126]. Across all of these indications, the recurrent and unifying conclusion is that liposomal encapsulation does not merely replicate the therapeutic effect of the free drug at a lower dose; it qualitatively changes the pharmacokinetic profile at the target tissue in a way that conventional vehicles cannot, making surface-engineered liposomes a platform technology rather than a disease-specific solution.

#### 3.9.1. Clinical Translation Roadmap for Surface-Modified Ocular Liposomes

**Figure 7:**
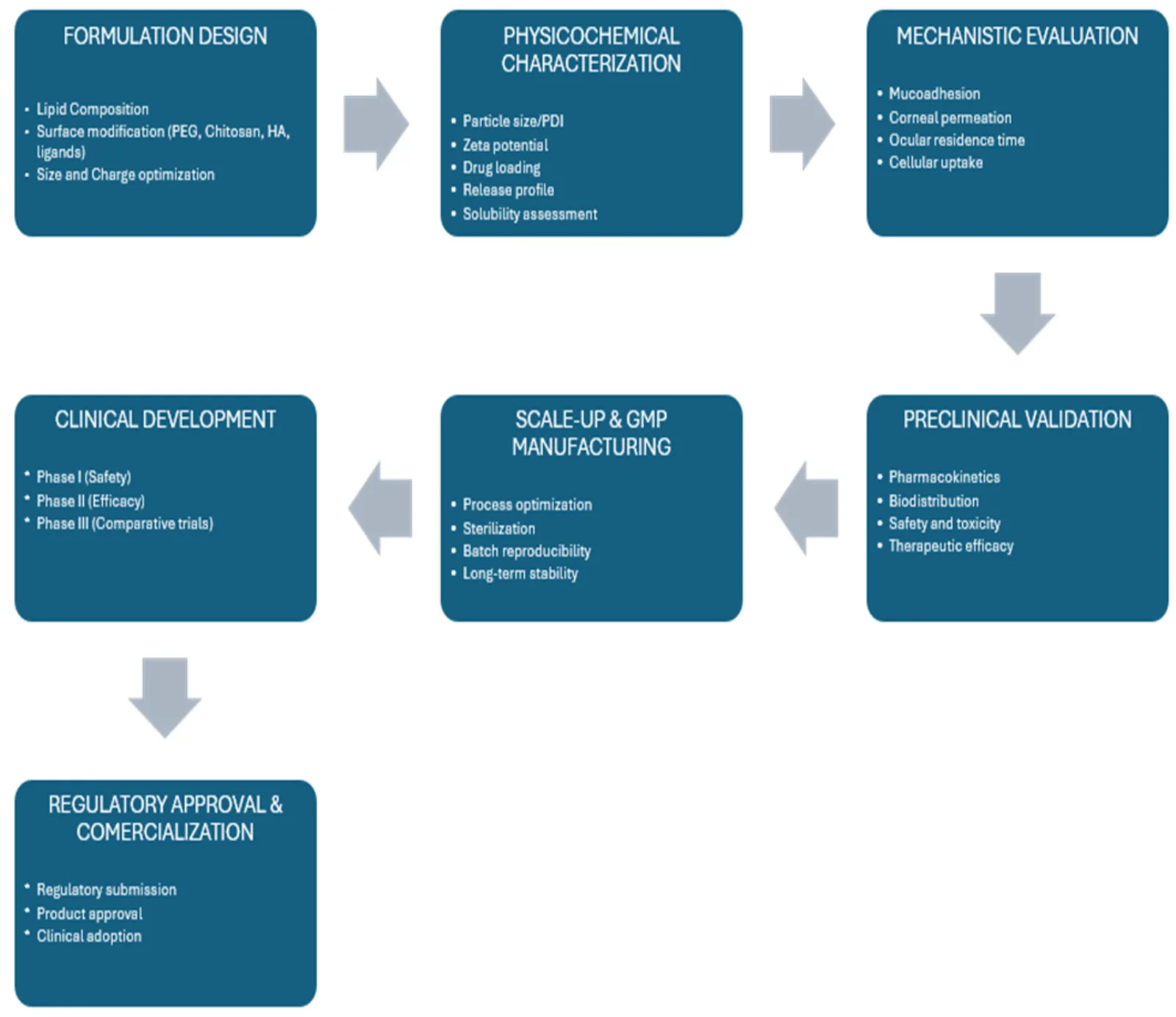
Schematic representation of step-by-step approach from bench to bedside for liposomal ophthalmic formulations.

The clinical translation of surface-modified ocular liposomes begins with a rational formulation design, including the optimization of lipid composition, particle size, surface charge, and functional coatings to enhance corneal penetration and retention. This is followed by mechanistic and preclinical studies evaluating permeability, pharmacokinetics, efficacy, and ocular safety. Successful formulations must then undergo scalable GMP manufacturing, sterilization validation, and long-term stability testing before advancing through clinical trials and regulatory approval. A schematic representation of a step-by-step approach from bench to the bedside is shown below (Figure 7). However, the major challenges to this translation include maintaining formulation stability, sterility, drug leakage from liposomes, ensuring batch-to-batch reproducibility, developing cost-effective manufacturing processes, demonstrating clear clinical advantages over existing ophthalmic therapies and regulatory issues. The success of the formulation depends on several critical variables that influence product performance, stability, manufacturability, and overall quality. The key formulation variables considered in the development of liposomal drug delivery systems for ophthalmic use are summarized below in Table 10.

The Table 11 highlights the key design principles that drive the overall solution strategy and decision-making process for developing liposomal drug delivery systems for ophthalmic use.

#### 3.9.2. Emerging Technologies in Liposomal Ophthalmic Drug Products

There is ongoing research on some of the emerging technologies in liposomal ophthalmic drug delivery that have features that are missing from the conventional liposomal drug delivery system. Over time, these improved liposomal technologies have the potential to provide a special release feature, more efficient drug delivery, higher drug loading and versatility. Table 12 shows emerging technology in liposomal ophthalmic drug delivery systems. Collectively, these nine technology mentioned below push liposomal ocular delivery from passive, fixed-release carriers toward systems that release cargo on cue (stimuli-, ROS-, and enzyme-responsive designs), carry genetic rather than small-molecule payloads (RNA-LNPs), combine platforms for sustained depot release (liposome-hydrogel and exosome-liposome hybrids), and are designed and manufactured more predictively (AI-assisted optimization and QbD).

#### 3.9.3. Safety Considerations of Surface-Modified Liposomal Ocular Drug Delivery Systems

While surface-modified liposomes offer significant advantages for improving ocular bioavailability and corneal penetration, safety remains a major consideration during formulation development and clinical translation [141,142]. The eye is a highly sensitive organ, and strategies designed to increase corneal permeation or prolong ocular residence time may unintentionally affect the integrity of ocular tissues. Consequently, safety assessments must address ocular irritation, epithelial toxicity, inflammatory responses, cationic lipid-associated toxicity, and the effects of long-term or repeated administration [142].

#### 3.9.4. Ocular Irritation and Local Tolerability

Ocular irritation is one of the first safety parameters evaluated for any topical ophthalmic formulation. Patients may experience symptoms such as stinging, burning, redness, excessive tearing, foreign-body sensation, or transient blurred vision. Although liposomes are generally considered biocompatible because they are composed of phospholipids like those found in biological membranes, certain surface modifications can increase the risk of irritation [141,142].

For example, cationic liposomes, bile salts, and surfactant-containing transferosomes are specifically designed to interact more strongly with the ocular surface to enhance drug absorption. While these interactions improve retention and permeability, excessive interaction with corneal epithelial cells may disrupt membrane integrity and trigger irritation. Therefore, the concentration of permeation enhancers and the magnitude of positive surface charge must be carefully optimized to balance efficacy with tolerability.

#### 3.9.5. Corneal and Conjunctival Epithelial Toxicity

The corneal epithelium serves as the eye’s primary protective barrier and is particularly vulnerable to damage from highly interactive drug delivery systems. Many surface-engineering approaches intentionally modify epithelial tight junctions or membrane fluidity to improve drug penetration. Although these effects are often reversible, excessive or prolonged disruption may compromise barrier function [143].

Potential manifestations of epithelial toxicity include the loss of epithelial cell viability, alterations in tight-junction protein, increased corneal permeability, delayed epithelial healing, and enhanced susceptibility to infection [143].

Several studies discussed in this review reported minimal cytotoxicity for PEGylated, hyaluronic acid-coated, and chitosan-coated liposomes. However, because many investigations evaluated only short-term exposure, further research is needed to determine whether repeated administration could lead to cumulative epithelial damage over time.

#### 3.9.6. Cationic Lipid Toxicity

Cationic lipids are among the most effective tools for enhancing ocular drug delivery because they promote strong electrostatic interactions with the negatively charged mucin layer and corneal surface. However, they also represent one of the most significant sources of nanoparticle-associated toxicity [128,143].

Commonly used cationic lipids such as DOTAP, DDAB, and spermine derivatives may induce cellular stress through several mechanisms, including membrane destabilization, oxidative stress, mitochondrial dysfunction, and the activation of apoptotic pathways. In general, toxicity tends to increase as surface-charge density increases [128,141].

Although the reviewed studies demonstrated good ocular tolerability for many cationic formulations, the risk–benefit balance becomes particularly important for chronic therapies such as glaucoma, dry eye disease, and age-related macular degeneration, for which frequent dosing may be required for months or years. Strategies such as partial PEGylation, the incorporation of neutral phospholipids, or the optimization of cationic lipid content may help reduce these risks while maintaining enhanced bioavailability.

#### 3.9.7. Inflammatory and Immune Responses

Because the ocular surface contains resident immune cells and tightly regulated inflammatory pathways, nanocarrier systems have the potential to induce local immune responses. Depending on the formulation characteristics, surface-modified liposomes may stimulate inflammatory signaling through direct interactions with epithelial cells or immune cells. Potential inflammatory outcomes include an increased production of pro-inflammatory cytokine, conjunctival hyperemia, corneal edema, the recruitment of inflammatory cells and ocular discomfort [144].

Interestingly, many liposomal formulations reviewed in this article reduced inflammatory biomarkers because they enhance the delivery of anti-inflammatory therapeutics. Nevertheless, the intrinsic inflammatory potential of the carrier itself should not be overlooked. This is particularly relevant for highly cationic systems, dendrimer-coated liposomes, and ligand-targeted formulations that may alter cellular uptake pathways or immune recognition.

#### 3.9.8. Repeated-Dose and Chronic Administration Safety

Perhaps the most important unanswered question in ocular nanomedicine involves long-term safety. Many ocular diseases targeted by liposomal formulations are chronic conditions requiring lifelong therapy. Consequently, repeated-dose safety may be more clinically relevant than acute toxicity [144]. Potential risks associated with chronic administration include progressive epithelial damage, the persistent disruption of tight junctions, tear-film instability, chronic low-grade inflammation, the accumulation of carrier materials within ocular tissues, altered corneal sensitivity and changes in conjunctival morphology.

Although most studies report favorable short-term safety profiles, the majority evaluate treatment durations ranging from days to a few weeks. Long-term investigations examining repeated administration over several months are still limited. Such studies will be essential to establish the safety profile necessary for regulatory approval and widespread clinical adoption [144].

Encouragingly, several studies that were reviewed reported the preservation of the retinal structure and function, with no detectable retinal inflammation following treatment with ligand-targeted or dendrimer-modified liposomes. However, long-term retinal biodistribution and clearance studies remain scarce and warrant further investigation.

#### 3.9.9. Future Safety Considerations

The future development of ocular liposomal systems should incorporate comprehensive safety testing that extends beyond conventional acute irritation studies. Important endpoints should include corneal and conjunctival histopathology, cytokine and inflammatory marker profiling, tight-junction integrity assessment, oxidative stress evaluation, and long-term tolerability monitoring [141,143].

Overall, liposomal drug delivery systems possess a favorable safety profile and are generally better tolerated than many conventional permeation enhancers used in ophthalmic formulations. Nevertheless, safety is highly dependent on the formulation composition, surface charge, and dosing frequency. While surface modifications such as cationic charge engineering, dendrimer coating, bile-salt incorporation, and tight-junction modulation can substantially improve ocular bioavailability, these approaches also introduce potential risks related to epithelial toxicity, inflammation, and cumulative tissue exposure. Therefore, successful clinical translation will require careful optimization of formulation parameters to achieve an appropriate balance between enhanced therapeutic efficacy and long-term ocular safety.

#### 3.9.10. Industrial Challenges in Liposomal Ophthalmic Formulation Productions

Good Manufacturing Practice (GMP): The liposomal ophthalmic formulation at an industrial scale must adhere to strict GMP regulations, which implies strict requirements for sterility testing, terminal sterilization, aseptic manufacturing, endotoxin levels, process control and quality control. In the small-scale manufacturing of liposomes, organic solvents such as chloroform are used, which must be avoided in large-scale GMP manufacturing due to toxicity. Also, strict requirements for documentation and validation during manufacturing meet the requirement of a consistent, predefined specification of products. Solvent or buffer precipitation issues at a large scale must be resolved under regulatory guidelines. The process must be validated to ensure that the trace solvent and byproducts are within the regulatory limit [145].

Quality control: The industrial scale-up of liposomal ophthalmic formulations is limited by batch-to-batch variability, quality control, the high cost of manufacturing and the complex process of manufacturing. The addition of organic solvents in the synthesis of liposomes adds another layer of complexity to the process. Scaling up and manufacturing reproducibility are difficult to achieve in liposomal ophthalmic formulations, as the liposomal system requires a precise lamellarity particle size, encapsulation efficiency, and a release profile, which could be achieved in small batches by optimizing thin-film hydration and microfluidics approach. But in large-scale manufacturing, variation in bilayer formation, drug loading, and vesicle stability is very common. Microfluidics and super-critical fluidics can help with the scale-up process and enhance reproducibility. Research on advanced manufacturing techniques like 3D printing and hot melt extrusion is being conducted to overcome some of the reproducibility issues; however, these methods are still in progress in the field of ocular formulation [146].

Long-term storage: Lipids in liposomes are inherently prone to the oxidation, hydrolysis, aggregation and leakage of drugs, leading to instability and degradation during storage. Long-term liposome stability requires controlling oxidation and hydrolysis, which can be achieved by incorporating antioxidants and using buffer systems that protect lipid integrity. Aqueous dispersions can be stored at 4 °C for short periods (about 1 month to 1 year). For extended storage, cryoprotectants such as sucrose or trehalose enable frozen preservation at −80 °C to −150 °C. Lyophilization with cryoprotectants extends the shelf life for 3-5 years by preserving liposomal integrity during storage [145].

Regulatory approval: The regulation of liposomal ophthalmic products is again cumbersome, as are ophthalmic formulations for topical or local drug delivery, defined as one type of complex product by the US FDA, which makes it more challenging for chemistry, manufacturing, and control (CMC) during the drug development of these products as compared to conventional ones. The FDA’s 2023 Quality Considerations for Topical Ophthalmic Drug Products provides a requirement for topical ophthalmic drug products for manufacturers https://www.fda.gov/regulatory-information/search-fda-guidance-documents/quality-considerations-topical-ophthalmic-drug-products (accessed on 10 August 2026). The US FDA has strict requirements for CMC characterization, in vitro release, IVIVC and in vivo pharmacokinetics studies in different segments of the eye. However, there is still the possibility of improving liposomal ophthalmic drug delivery by collaborating with industry and regulatory bodies to adopt a standardized protocol for liposomal physicochemical characterization and quality control, which can ease regulatory approval [147].

Sterilization: For ophthalmic formulations, there are strict requirements for ophthalmic solutions to be sterilized. Since liposomal formulations are unstable at the high temperature of an autoclave, terminal sterilization with an autoclave is not possible; instead, bulk solutions are sterilized using filters, typically 0.2 μm filters, to remove microbial contaminants, and then they are filled aseptically in the final container. To maintain aseptic conditions, equipment like clean-in-place (CIP) and steam-in-place (SIP) systems attached to reactors and piping are being used [145]. The finished product must undergo a sterility test.

Batch consistency and reproducibility: The liposomal ophthalmic formulations have stringent requirements for batch-to-batch consistency and reproducibility. It is important to produce liposomes of equal size distribution, drug loading, and release profile consistently across each production batch. A little deviation in process parameters can affect liposomal composition. Therefore, automated processes and in-line monitoring are applied to maintain product uniformity [145].

Cost considerations: The overall cost of making liposomal ophthalmic products is higher compared to the conventional ophthalmic formulations. This is primarily due to the high cost of aseptic manufacturing techniques, specialized equipment such as high-pressure homogenizers, microfluidics and their cost of operation and ocular safety considerations [145]. Costs also add up through the use of highly expensive pharmaceutical-grade lipids and cholesterol. With surface-modified liposomes, this cost increases further. Losses during the scale-up process also add to the costs. Liposomal ophthalmic products also have strict quality control requirement for particle size, encapsulation efficiency, endotoxin, sterility testing and stability testing, which further elevate the costs. Lyophilization for dried formulations and cold storage requirements for aqueous liposomes also add additional costs for equipment and resources [146].

## 4. Challenges and Future Perspectives

While significant progress has been made in the development of liposomal ocular drug delivery, there are still several challenges that prevent its broader clinical translation. The fundamental constraint of drug-loading capacity, especially for hydrophilic drugs in lipid bilayers, still exists. Sterilization without causing damage to the vesicles is a challenge in formulation, particularly for thermosensitive lipid composition. There are many other challenges that still exist for liposomal delivery, including long-term colloidal stability during storage, the susceptibility to aggregation or leakage of the drugs, and scaling up the manufacturing process [148]. In an extensive survey article, Lacrisek et al. provided additional background on these limitations, highlighting the fact that the most clinically mature nanocarrier class is the liposomal system, but large-scale production and regulatory-grade sterilization processes are still important to further expedite clinical translation [69]. The industrial translation of liposomal ophthalmic formulations and drug delivery systems remains more challenging than conventional ophthalmic formulations, owing to the structural sensitivity of liposomes and stringent quality and sterility requirements for ocular administration. Scaling up and manufacturing reproducibility are difficult to achieve in liposomal ophthalmic formulations, as a liposomal system requires a precise lamellarity particle size and encapsulation efficiency, which could be achieved in small batches by optimizing the thin-film hydration and microfluidics approach. But in large-scale manufacturing, variation in bilayer formation, drug loading, and vesicle stability is very common. Advanced manufacturing like 3D printing and hot-melt extrusion are being studied to overcome some of the reproducibility issues; however, these methods are still in progress in the field of ocular formulations. Also, sterility requirements for ocular products are very stringent, as liposomes are very sensitive to autoclaves and gamma radiation.

The regulation of liposomal ophthalmic products is cumbersome, as ophthalmic formulations for topical or local drug delivery are defined as one type of complex product by the US FDA, which makes it more challenging for the chemistry, manufacturing, and control (CMC) during the drug development of these products as compared to conventional ones. The FDA’s 2023 Quality Considerations for Topical Ophthalmic Drug Products provides requirements for topical ophthalmic drug products for manufacturers. The US FDA has strict requirements for CMC characterization, in vitro release, IVIVC and in vivo pharmacokinetics studies in different segments of eye. For ocular liposomes, regulators require additional biocompatibility, irritation, and immunogenicity testing due to the sensitivity of ocular tissues.

In the future, the use of stimuli-responsive liposomal systems such as light-responsiveness, pH-responsiveness and thermos-responsive vesicles is expected to open interesting opportunities for the spatiotemporal control of drug release at specific ocular tissues. The combination of liposomal carriers with gene therapy and biologics and combination drug treatments is an exciting area, especially for those diseases of the posterior segment that are complex, including AMD, DR, and inherited retinal dystrophies. The ongoing development of microfluidic fabrication technologies for liposome preparation is anticipated to lead to further improvements in the precision of particle size, lamellarization, and surface functionalization, thereby enhancing the reproducibility of particles produced across different batches and the potential for clinical translation. In vitro–in vivo correlation (IVIVC) models specific to the eye will also be crucial in the rational design and regulatory assessment of future generations of liposomal systems. Surface-engineering techniques are becoming increasingly sophisticated, and the potential clinical applications of engineered liposomes in ophthalmology are likely to grow substantially in the future.

## 5. Conclusions

Liposomal drug delivery systems represent a transformative platform in ocular therapeutics, offering a structurally versatile and clinically translatable approach to overcome the major barriers that limit conventional ophthalmic formulations. Through surface-engineering strategies, liposomes can be rationally designed to improve ocular drug delivery, including cationic charge modification, PEGylation, mucoadhesive polymer coatings, bile-salt incorporation, dendrimer functionalization, and ligand-based targeting. These modifications enhance electrostatic interactions with the ocular surface, extend precorneal residence times, improve transcorneal permeation, and enable controlled and sustained drug release across both anterior and posterior-segment targets. The clinical trajectory of liposomal ophthalmic systems, as evidenced by FDA-approved products such as Visudyne^®^, Lacrisek^®^, and Artelac Rebalance^®^, and a growing pipeline of clinical-stage candidates and patent-protected innovations, reinforces the therapeutic credibility of this approach. The synthesis of robust preclinical data with emerging clinical evidence positions engineered liposomes as not merely experimental constructs but genuinely viable, patient-centered solutions to the long-standing challenges of ocular drug delivery and targeting.

### Search Strategy and Selection Criteria

A structured literature search was performed using scientific databases such as PubMed, Scopus and Web of Science, NCBI, and Google Scholar. The articles cited in this review were published from 1999 to 2026. Keywords related to liposomal surface-modification strategies, nanocarrier-based therapeutics, and challenges in liposomal ophthalmic delivery were used to identify relevant studies. Manuscripts with original experiments, preclinical or clinical data and review articles with primary data were included. For clinical data information, clinicaltrials.gov was also searched for each clinical study. Review articles lacking primary data and non-ophthalmic liposomal applications were excluded.

## Figures and Tables

**Table 1:** Classification of ocular barriers in drug delivery based on anatomical segment.

Segment	Static Barriers	Dynamic Barriers	Intraocular Barriers
Anterior	Cornea and conjunctiva (Bowman’s layer, Descemet’s membrane); Stroma; Endothelium	Tear film; Blinking; Nasolacrimal drainage	Blood–aqueous barrier (BAB)
Posterior	Vitreous body; Sclera and Bruch’s membrane	Conjunctival blood flow; Lymphatic drainage	Blood–retinal barrier (BRB)

**Table 2:** FDA-approved liposomal ocular drug products and some commercially available eye care products.

Liposomal Ophthalmic Drugs	Disease/Condition	Drug Product	Status
Visudyne^®^	Wet age macular degeneration	Verteporfin	FDA-approved
Artelac Rebalance^®^	Dry eye disease	Vitamin B 12	Commercially available
Clinitas Hydrate^®^	Dry eye disease	Carbomer 980	Commercially available
Lacrisek^®^	Dry eye disease	Vitamin A, E	Commercially available

**Table 3:** Liposomal ocular formulations in clinical stage.

Formulation/Product	Nanocarrier Type	Drug/Component	Indication	Status/Year
Liposomal latanoprost	Liposomes	Latanoprost	Ocular hypertension/glaucoma	Phase I/II (NCT01987323)
TLC399 (ProDex)	Liposomes	Dexamethasone prodrug	Diabetic macular edema	Phase II (NCT03093701)
LAMELLEYE	Liposomes	Phospholipid formulation	Dry eye disease	Not yet available as an Over-the-counter product
POLAT-001	Liposomes	Latanoprost	Ocular hypertension/open-angle glaucoma	Phase II (NCT02466399)
Hyaluronic acid liposomes	Liposomes	Hyaluronic acid	Meibomian gland dysfunction	Clinical trial (NCT03617315)
(OZODROP^®^)	Liposomes	Ozone-based solution	Ocular infection	Phase IV NCT04087733
Liposomal sirolimus	Liposome	Sirolimus	Dry Eye Disease	Phase I NCT04115800

**Table 4:** Representative patents for liposomal ocular drug delivery systems.

Patent	Liposomal Strategy	Drug	Route	Indication	Key Outcome
US9956195B2	Prostaglandin-loaded liposomal formulation	Prostaglandin F2α	Subconjunctival injection	Glaucoma/elevated IOP	Sustained release enabling IOP reduction for ~4–6 months
US10272040B2	Liposomal ocular formulation	Latanoprost	Subconjunctival injection	Glaucoma/ocular hypertension	~60% of the drug release sustained over ~10 days
CN109906075A	Corticosteroid-loaded liposomes	Corticosteroid	Local ocular injection	Ocular inflammation	Targeted delivery to inflamed ocular tissues

**Table 5:** Comparative summary of surface-modification strategies.

Strategy	Refs.	Primary Mechanism	Representative Reported Enhancement	Cross-Study Reproducibility	Key Trade-Off
Cationic surface charge (Section 3.2)	[14,74,75,76,77,78,79,80,81,82,83]	Electrostatic adsorption to anionic mucin/corneal surface	Corneal drug levels/AUC ~3- to 5-fold higher [75,76]; Papp ~2-fold higher [82]	High—~10 independent studies, 8 drug classes, consistent direction and magnitude	Charge-density-dependent cytotoxicity/inflammation on chronic dosing
PEGylation (Section 3.3)	[16,17,18,60,84,85,86,87]	Steric stabilization; reduced opsonization/enzymatic degradation; mucus-penetrating	Improves posterior retention/half-life more than early corneal permeation; TPGS-PEG Papp +10.2-fold vs. commercial [87]	Moderate—time- and tissue-dependent; most reliable when paired with muco-adhesion [108]	Alone, does not reliably increase early corneal permeation
Mucoadhesive coating—chitosan/HA/HAMA (Section 3.4)	[18,88,89,90,91,92,93,94]	Electrostatic + covalent (HAMA thiol–Michael) mucin binding; reversible tight-junction modulation	Cumulative corneal permeation 35% to 65–70% [90] AUC +3.9-fold, Papp +3.18-fold [88]	High—~9 independent studies; covalent > electrostatic > uncoated ordering reproduced	Added synthesis complexity; larger particle size (up to ~285 nm)
Bile salts/bilosomes (Section 3.5)	[95,96,97,98]	Membrane fluidization + Ca2+-mediated tight-junction opening + micellar solubilization	Papp +3.7 to +4.5-fold vs. cholesterol liposomes [95]; atenolol/timolol permeation +5.2 to +5.8-fold	High in magnitude, moderate in safety—effect reverses above CMC [114]	Narrow, concentration-dependent therapeutic window
Dendrimer coating—PAMAM (Section 3.6)	[99,100,101,102,103,104,105,106]	Surface-charge tuning + electrostatic drug entrapment; antioxidant protection at retina	Higher Cmax, prolonged residence, retinal protection reported [99,102,103,104,105,106]	Low—single formulation system; not yet independently replicated	Narrow evidence base limits generalizability
Ligand targeting/CPPs (Section 3.7)	[107,108,109,110,111,112]	Receptor-mediated endocytosis (Tf, Tet1, alpha-2 agonist) or CPP-driven uptake (TAT, penetratin)	Retinal concentration +14-fold (TAT-RGD); eye-to-retina transport 1.23% to 3.66% [112]	Moderate—largest posterior gains, but effect size is ligand-specific, not generalizable	Highest formulation complexity; immunogenicity risk (peptide/antibody ligands)
Edge activators/transferosomes (Section 3.8)	[109,113]	Vesicle deformability via single-chain surfactant edge activators; trans-pore hydrotaxis	Conjunctival penetration +4-fold, cornea ~2-fold vs. oily control [109]; natamycin Papp +9 to +16-fold [113]	Moderate—consistent tissue hierarchy reproduced across ~2–3 studies	Favors conjunctival-scleral route more than the cornea itself

**Table 6:** Comparative analysis of several surface-modified strategies of liposomal ophthalmic formulation with respect to complexity, safety margin, efficacy and reproducibility.

Strategy	Efficacy	Reproducibility	Safety	Complexity
Cationic surface charge	High	High	Moderate	Low
PEGylation	Moderate	Moderate	High	Low
Mucoadhesive coating	High	High	High	Moderate
Bile salts/bilosomes	High	Moderate	Moderate	Low
Dendrimer coating (PAMAM)	Moderate	Low	Moderate	High
Ligand targeting/CPPs	High	Moderate	Moderate	High
Edge activators/transferosomes	Moderate	Moderate	High	Moderate

Legend: green = favorable/high · yellow = moderate/condition-dependent · red = limited/needs more evidence.

**Table 7:** Reproducibility of the reported results.

Strategy	Independent Studies Cited	Effect-Direction Consistency	Effect-Magnitude Consistency
Cationic surface charge	~10	Consistent (10/10)	Consistent (~2- to 5-fold range across drugs)
PEGylation	4	Consistent alone; reliably positive only when combined with mucoadhesion	Variable (time- and tissue-dependent)
Mucoadhesive coating	~9	Consistent (9/9)	Consistent (covalent > electrostatic > uncoated)
Bile salts/bilosomes	4	Consistent for efficacy; concentration-dependent for safety	Variable (~2- to 4.5-fold, salt-dependent)
Dendrimer coating (PAMAM)	1	Consistent within the single system reported	Not yet independently tested
Ligand targeting/CPPs	~5	Consistent (5/5)	Highly variable (ligand/receptor-dependent)
Edge activators/transferosomes	~2-3	Consistent	Variable (drug- and gel-matrix-dependent)

**Table 8:** A comparative analysis of different surface-modification strategies in terms of the mechanism of corneal penetration, corneal permeability, ocular residence time, drug-loading, safety and clinical translational status.

Strategy	Mechanism of Corneal Penetration	Corneal Permeability	Ocular Residence Time	Drug Loading/Stability	Safety	Manufacturing Complexity	Clinical Translation Status	Ref
**Cationic Liposomes**	Electrostatic interaction with negatively charged mucin and corneal epithelium; enhanced endocytosis	High (typically 2–5 fold enhancement)	High	Good encapsulation efficiency and moderate colloidal stability	Excessive positive charge may cause irritation or epithelial toxicity	Low-Moderate	Most clinically mature; basis of Novasorb^®^ technology and several ophthalmic products	[14,74,75,76,77,78,79,80,81,82,83]
**PEGylated Liposomes**	Improved mucus penetration; steric stabilization; prolonged tissue retention	Moderate	Moderate-High	Excellent physical stability and reduced aggregation	Generally safe; may reduce cellular interaction if over-PEGylated	Low	High translational potential due to extensive regulatory experience with PEGylated nanomedicines	[16,17,18,60,84,85,86,87,88]
**Chitosan-Coated Liposomes**	Muco-adhesion with transient opening of corneal tight junctions	High	Very High	Improved stability and sustained release	Generally biocompatible; concentration-dependent irritation possible	Moderate	Strong translational promise for topical products	[88,89]
**HA/HAMA-Coated Liposomes**	Muco-adhesion, CD44 receptor interactions, prolonged precorneal retention	High	Very High	Good stability and controlled release	Excellent ocular tolerability	Moderate	Attractive for dry eye and posterior-segment delivery applications	[88,89,90,91,92,93,94]
**Bilosomes (Bile-Salt Liposomes)**	Membrane fluidization,	Very High	Moderate	High encapsulation efficiency; improved membrane flexibility	Bile-salt concentration must be optimized to avoid epithelial damage	Moderate	Preclinical stage with strong permeation data	[61,62,63,64]
	vesicle							
	deformability, transient loosening of epithelial tight junctions							
**Dendrimer-Modified Liposomes**	Enhanced cellular uptake and electrostatic interactions, improved intracellular delivery	High	Moderate-High	Very high loading potential and surface functionality	Surface-charge-related toxicity requires careful control	High	Early translational stage; promising for retinal delivery	[99,100,101,102,103,104,105,106]
**Ligand-Targeted Liposomes**	Receptor-mediated endocytosis and active tissue targeting	High and tissue-specific	Moderate-High	Variable; depends on ligand conjugation strategy	Generally safe but requires extensive biological validation	High	Precision-medicine approach; largely preclinical/early translational	[107,108,110,111,112]
**Transferosomes (Edge Activator-Modified Liposomes)**	Extreme vesicle deformability enables passage through narrow intercellular spaces	Very high	Moderate	Good drug loading, but long-term stability can be challenging	Well tolerated	Moderate-High	Strong preclinical evidence for anterior and posterior-segment delivery	[109,113]

**Table 9:** Advantages and disadvantages of various surface-modification strategies for liposomes.

Surface-Modification Strategies	Advantage	Disadvantage
Cationic liposomes	Long-lasting adhesion to the ocular surface due to strong electrostatic interaction with negatively charged mucin. Longer pre-corneal residence time and better trans corneal permeation of the drug due to positive charge and higher internalization. Excellent corneal absorption over neutral or anionic liposomes.	Excessive binding to mucin leads to tear-film destabilization and blurred vision. Not suitable for chronic eye disease due to irritation. Regulatory consideration: Stringent requirement for safety due to irritation, safety and stability concerns. Poor tolerance for topical intravitreal or subconjunctival use. High immunogenicity and inflammation.
PEGylation	Provides hydrophilic stearic hinderance and enhances colloidal stability, reduces protein adsorption, leading to lower immunogenicity and enzymatic degradation. Longer precorneal residence compared to non-pegylated liposomes. Longer shelf life due to less aggregation.	Excessive Pegylation may increase clearance by blinking and tears. High PEG density may lead to blurred vision. PEGylated lipids are costlier as compared to conventional liposomes, require tight control of PEG density. May require cold chain storage due to PEG being used.
Mucoadhesive Polymer coatings	Strong interaction with mucin layer of tear film and conjunctiva enhances the precorneal residence time as well as corneal permeability. Colloidal stability is due to presence of polymer coating, and less aggregation. Controlled or prolonged drug release. Biocompatible polymers such as chitosan and Hyaluronic acid (HA) reduce inflammatory response.	Cationic polymers like chitosan may cause mild irritation in the eye. Presence of polymer layers may add difficulty in GMP manufacturing and batch reproducibility. Use of additional excipients increases the CMC burden, cost and sterility requirement.
Bile-salt incorporation (Bilosomes)	Bile salt acts as a permeation/penetration enhancer by inducing membrane fluidity and loosening of tight-junction thereby increasing corneal and conjunctival permeability. Better loading capacity and improves the solubility. Controlled drug release possible.	Excessive membrane fluidization may lead to epithelial disruption due to membrane fluidity, stability issue and premature leakage can occur. High bile-salt concentration may cause lipid lysis and destabilize lipid and promote aggregation. Since bile salt is detergent it can induce ocular-surface inflammation represented as stinging and burning with repeated dosing. GMP manufacturing is cumbersome as stringent requirement for bile-salt concentration and removal of bile salt. Regulatory requirement: More irritation and safety studies needed.
Dendrimer -assisted surface modification	Dendrimers (PAMAM) increase drug-loading capacity due to inherent higher cargo capacity of dendrimers. Increase residence time due to presence of surface-modification group at dendrimers.	Cationic dendrimer (PAMAM G3-G7) causes irritation in eye and may induce immunogenicity. Complex structure and synthesis may lead to batch-to-batch variability and require more quality control. Higher cost.
Ligand-targeted surface modification	Ligands such as antibodies, peptides, receptor-specific ligands lead to target specific binding to receptors. Cellular uptake occurs via receptor-mediated endocytosis and leads to higher drug concentration by internalization at cornea, conjunctiva and retina. Higher drug concentration at the target site. Since the binding is specific to ocular tissue, less off-target toxicity.	Ligands such as peptides and antibodies may induce inflammation and immune reactions. Complex ligand chemistry can increase the CMC, increase cost, more quality control, high batch-to-batch variability.
Edge Activators modified liposomes (Transferosomes)	Edge activators such as Tween 80, span 80, sodium cholate incorporated in the phospholipid bilayer cause extreme membrane flexibility and deformability enabling penetration through tight junction of corneal and conjunctival permeation. Higher bioavailability compared to traditional liposome due to improve trans corneal and trans conjunctival transport.	Excessive deformability may lead to premature leakage of drug from phospholipid bilayer. Surfactant content can destabilize the vesicle and alter the loading encapsulation. Irritation or discomfort possible due to surfactants and some edge activators may cause burning and stinging, dose-dependent toxicity, epithelial disruption and inflammation. GMP is complex. Tight surfactant control, batch variability.

**Table 10:** Formulation variables considered in development of liposomal ophthalmic formulations.

Formulation Variable	Description	Examples and Ranges Used	Effect on Liposome Properties	Impact on Ocular Delivery and Performance
**Phospholipid Composition**	Type and mixture of phospholipids forming the bilayer	DSPC, DPPC, HSPC, EPC, DOPC, POPC	Determines membrane packing, permeability, and phase transition temperature (Tm)	Influences drug retention, corneal interaction, stability, and circulation half-life
**Lipid Saturation**	Degree of saturation of acyl chains	Saturated (DSPC, DPPC) vs. Unsaturated (DOPC, POPC)	Saturated lipids form rigid, less permeable bilayers; unsaturated lipids increase fluidity	Affects drug leakage, membrane fusion, and biodistribution
**Acyl Chain Length**	Length of hydrocarbon chains in phospholipids	C14-C18-C22	Longer chains increase hydrophobic interactions and membrane stability	Improves retention of encapsulated drugs and structural integrity
**PEG Density (PEGylation Level)**	Surface coverage by polyethylene glycol chains	1–10 mol% DSPE-PEG2000	Creates steric barrier that reduces protein adsorption and aggregation	Increases circulation half-life and reduces RES uptake
**PEG Chain Length**	Molecular weight of PEG attached to lipids	PEG1000-PEG5000	Longer PEG chains enhance steric stabilization but may hinder cellular uptake	Improves “stealth” behavior but may reduce target-cell interaction
**Cholesterol Content (% mol)**	Cholesterol incorporated into lipid bilayer	20–50 mol%	Reduces membrane permeability, increases packing density and mechanical stability	Improves stability in tear fluid
**Bilayer Fluidity**	Mobility of lipids within bilayer	Low to high fluidity depending on composition	Controls membrane permeability, deformability, and fusion characteristics	Influences drug release kinetics, membrane interaction, and tissue penetration
**Particle Rigidity**	Mechanical stiffness of vesicle membrane	High in saturated/cholesterol-rich bilayers	More rigid vesicles resist deformation and leakage	Affects corneal penetration
**Particle Size**	Mean hydrodynamic diameter	50–200 nm (common therapeutic range)	Affects surface area, drug loading, and clearance rate	One of the most important factors for ocular penetration
**Polydispersity Index (PDI)**	Measure of size distribution	<0.2 preferred	Reflects formulation homogeneity	Lower PDI improves reproducibility and predictable pharmacokinetics
**Surface Charge (ζ-Potential)**	Net electrical charge at particle surface	Negative (−30 mV), Neutral, Positive (+30 mV)	Govern colloidal stability and interactions with biological membranes	Particularly important because the ocular mucin layer is negatively charged
**Lamellarity**	Number of lipid bilayers	SUV, LUV, MLV	Determines internal aqueous volume and drug-loading capacity	Affects encapsulation efficiency and release kinetics
**Drug-to-Lipid Ratio**	Amount of encapsulated drug relative to lipid content	Formulation dependent	Influences loading efficiency and membrane integrity	Determines therapeutic efficacy and toxicity profile
**Phase Transition Temperature (Tm)**	Temperature at which bilayer changes from gel to liquid-crystalline state	DPPC: ~41 °C; DSPC: ~55 °C	Controls membrane fluidity and permeability at physiological temperature	Impacts drug retention and temperature-sensitive release
**Mucoadhesive Surface Modification**	Surface engineering using mucoadhesive polymers or cationic lipids	Chitosan, hyaluronic acid, Eudragit coatings, stearylamine, DOTAP; typically, 0.1–1% polymer coating or 5–20 mol% cationic lipid	Enhanced ocular-surface adhesion, prolonged retention, reduced tear washout, improved penetration, and sustained release	Increased precorneal residence time and ocular bioavailability

References: [73,127,128,129].

**Table 11:** Key design principles and desired formulation strategy for developing ophthalmic liposomal drug delivery systems.

Design Principle	Desired Formulation Strategy	Expected Outcome
Prolonged ocular residence	Slightly positive surface charge, PEGylation, or mucoadhesive coating	Reduced tear drainage and increased contact time
Enhanced corneal penetration	Small particle size (50–200 nm), flexible bilayer composition	Improved drug transport across the cornea
High drug retention	Saturated phospholipids + cholesterol	Reduced premature leakage during storage and administration
Sustained drug release	Multilamellar vesicles or rigid bilayers	Extended therapeutic effect and reduced dosing frequency
Low ocular irritation	Neutral or mildly positive surface charge, biocompatible phospholipids	Improved patient tolerance and safety
Maximum formulation stability	Optimized cholesterol content and low PDI (<0.2)	Reduced aggregation and enhanced shelf life
Sustained drug release	Multilamellar vesicles or rigid bilayers	Extended therapeutic effect and reduced dosing frequency

References: [73,127,128,129].

**Table 12:** Summary of emerging technologies and their application in ocular liposomal delivery.

Emerging Technology	Relevance to Ocular Liposomal Delivery	Reference(s)
**Stimuli-responsive liposomes (umbrella)**	Umbrella term for release triggered by pH, redox potential, reactive oxygen species (ROS), enzymes, temperature, or light—relevant where a single intravitreal dose must adapt its release rate to fluctuating disease activity over weeks rather than releasing at a fixed rate.	[130]
**Thermosensitive liposomes**	Lipid bilayer shifts from gel to liquid-crystalline phase at ~40–42 degC, releasing cargo on cue; a chitosan-coated thermosensitive liposome/in situ gel raised transcorneal metoprolol permeation 2.4-fold over the uncoated formulation in a glaucoma model.	[131]
**ROS-responsive systems**	Thioether- or boronate-ester-modified lipids oxidize and destabilize the bilayer in the elevated-ROS microenvironment of ocular inflammation, infection, or oxidative stress (e.g., dry eye, uveitis), giving disease-triggered rather than time-triggered release.	[132]
**Enzyme-responsive liposomes**	Ester or peptide linkers are cleaved by disease-elevated matrix metalloproteinases (MMPs), phospholipases, or esterases—relevant to corneal ulceration and infectious keratitis, where local enzyme activity is already pathologically elevated.	[133]
**Lipid nanoparticles (LNPs) for RNA delivery**	LNP-encapsulated messenger RNA (mRNA) or self-amplifying RNA (saRNA), delivered intravitreally, transfects Müller glia and other inner-retinal cells—opening a gene-expression route for inherited retinal disease that small-molecule liposomal delivery cannot address.	[134]
**Hybrid liposome-hydrogel systems**	Liposomes loaded within a thermo- or near-infrared (NIR)-responsive hydrogel depot combine liposomal drug-loading versatility with a hydrogel’s sustained, on-demand release—demonstrated for intravitreal retinoblastoma therapy and ocular-surface tumor therapy.	[135,136]
**Exosome-liposome hybrids**	Membrane fusion of natural exosomes with synthetic liposomes combines exosome biocompatibility and cell targeting with a liposome’s higher, more consistent drug-loading capacity, addressing the low-yield limitation of exosomes used alone.	[137]
**AI-assisted formulation optimization**	Machine-learning models trained on microfluidic production data predict liposome size, encapsulation efficiency, and flow-rate-ratio process parameters directly, reducing the trial-and-error screening burden of manual formulation development.	[138,139]
**Quality by Design (QbD)**	Risk-based, systematic formulation development—defining a Quality Target Product Profile (QTPP), identifying Critical Quality Attributes (CQAs), and using Design of Experiments (DoE)—applied specifically to ophthalmic liposomes, including a QbD-optimized caspofungin liposome for fungal keratitis.	[83,140]

## Data Availability

No new data were created or analyzed in this study. Data sharing is not applicable to this article.
